# Validation of CT-based ventilation and perfusion biomarkers with histopathology confirms radiation-induced pulmonary changes in a porcine model

**DOI:** 10.1038/s41598-023-36292-0

**Published:** 2023-06-09

**Authors:** Mattison J. Flakus, Antonia E. Wuschner, Eric M. Wallat, Melissa Graham, Wei Shao, Dhanansayan Shanmuganayagam, Gary E. Christensen, Joseph M. Reinhardt, John E. Bayouth

**Affiliations:** 1 Department of Medical Physics, University of Wisconsin - Madison, Madison, WI USA; 2 Research Animal Resources and Compliance, University of Wisconsin - Madison, Madison, WI USA; 3 Department of Medicine, University of Florida, Gainesville, FL USA; 4 Department of Surgery, University of Wisconsin - Madison, Madison, WI USA; 5 Department of Animal and Dairy Sciences, University of Wisconsin - Madison, Madison, WI USA; 6 Department of Electrical and Computer Engineering, University of Iowa, Iowa City, IA USA; 7 Department of Radiation Oncology, University of Iowa, Iowa City, IA USA; 8 Roy J. Carver Department of Biomedical Engineering, University of Iowa, Iowa City, IA USA; 9 Department of Radiation Medicine, Oregon Health Sciences University, Portland, OR USA

**Keywords:** Biomarkers, Pathogenesis

## Abstract

Imaging biomarkers can assess disease progression or prognoses and are valuable tools to help guide interventions. Particularly in lung imaging, biomarkers present an opportunity to extract regional information that is more robust to the patient’s condition prior to intervention than current gold standard pulmonary function tests (PFTs). This regional aspect has particular use in functional avoidance radiation therapy (RT) in which treatment planning is optimized to avoid regions of high function with the goal of sparing functional lung and improving patient quality of life post-RT. To execute functional avoidance, detailed dose–response models need to be developed to identify regions which should be protected. Previous studies have begun to do this, but for these models to be clinically translated, they need to be validated. This work validates two metrics that encompass the main components of lung function (ventilation and perfusion) through post-mortem histopathology performed in a novel porcine model. With these methods validated, we can use them to study the nuanced radiation-induced changes in lung function and develop more advanced models.

## Introduction

Lung cancer is one of the most commonly diagnosed and deadly cancers^1^. It is commonly treated using radiation therapy (RT). Unfortunately, radiation-induced lung injuries (RILI) are prevalent toxicities in 5–20% of lung cancer patients receiving RT and upwards of 33% of late-stage patients^1^. Early intervention and/or prevention of these toxicities is crucial to preserving patients’ quality of life post-treatment since these RILI can cause significant respiratory decline and even death^2,3^. To date, follow-up RILI diagnosis has primarily relied on pulmonary function tests (PFT) and Karnofsky performance status^4,5^. Both are methods that measure global lung information and show poor correlations with RILI prevalence^2,6–11^.

Imaging biomarkers have emerged as a non-invasive technique to provide information about regional variation in pulmonary function. Pulmonary function biomarkers also demonstrate robustness to patient variability. Several groups have developed techniques to estimate local lung function using computed tomography (CT)^12–19^, magnetic resonance imaging (MRI)^20,21^, and nuclear medicine imaging techniques^22–25^. Incorporating regional functional information into disease prediction models has been shown to increase their predictive power^26–28^. Beyond providing detailed regional disease progression information, pulmonary imaging biomarkers have been used for the application of functional avoidance RT. This RT treatment planning technique redirects radiation dose away from high-functioning lung regions (as defined by pulmonary biomarkers) to lower-functioning regions with the goals of preserving overall lung function and mitigating radiation-induced toxicities post-RT. In this process, treatment plans avoiding dose to the high functioning lung are generated using developed models predicting the pulmonary function response to delivered radiation dose^29,30^. Using imaging biomarkers, several groups have demonstrated the feasibility of sparing highly functional lung from high radiation dose^31–37^. Additionally, recent interim prospective clinical trial results suggest there may be a clinical benefit to doing so, but the results are not currently statistically significant^38^.

In moving toward widespread clinical implementation of functional avoidance planning techniques, one important step is developing more advanced predictive dose-response models incorporating nuances in the radiation response of pulmonary function. To achieve this goal, imaging biomarkers must be validated to confidently link imaging observations and physiological mechanisms of radiation response. To date, several studies have reported on different biomarker responses and clinical diagnoses^2,11,20,39^. However, most validation efforts have compared functional information between different imaging techniques^40^, and only a few studies have investigated lung pathology post-RT^41,42^. To our knowledge, no study has presented results of imaging biomarkers in conjunction with pathology findings to analyze radiation-induced pulmonary function changes. This work provides a thorough analysis of pulmonary radiation response from both imaging and histopathology observations and validates conclusions previously drawn from imaging techniques with tissue change observations. Validation between CT imaging and histopathology increases confidence in the accuracy of non-invasive CT-based pulmonary function assessments and takes an important step toward eventual clinical implementation. This study extensively compares biomarkers and pathology by including information about both aspects of pulmonary function (perfusion and ventilation) and evaluating regions receiving different dose levels. The purpose of this work was twofold: first was to measure pathological changes in the lung tissue of a porcine model to assess the radiation-induced change. Second was to compare these measurements to imaging biomarker measurements obtained from the same lungs that our group has reported previously. We hypothesized that the changes seen in the pathology would yield evidence of similar post-RT physiological change thus validating the use of the imaging biomarker methods as a reliable, non-invasive approach to assessing lung changes.

## Materials and methods

### Animal studies

Wisconsin Miniature Swine (WMS) is a human-sized breed with advantageous attributes for studying pulmonary radiation response. Previous work has detailed these advantages and demonstrated that WMS radiation response correlates well with that of humans^17,19,43^. This work reports results for perfusion and ventilation biomarkers and post-mortem histopathology for two groups of five WMS (ten total). These pigs (referred to as groups A and B) were overall evenly split between sexes to avoid any sex-dependent bias in results. Group A had three females / two males and group B had two females / three males. These subjects have been previously reported on for tasks related to assessing pulmonary function including developing perfusion imaging and radiation response methods^18,19,44^, demonstrating indirect ventilation response to radiation^17^, evaluating biomarker repeatability^45^, and image noise impact on ventilation biomarkers^46^. CT imaging, radiation treatment and biomarker derivation methods that are implemented in the current work have been described in detail in previous work^18,19,44–46^. Therefore, those methods are summarized and referenced in this work. Methodology emphasis is placed on aspects of this work related to histopathology, which has not been previously reported.

The WMS subjects used in this work were sourced by the Wisconsin Alumni Research Fund (WARF) and genetically engineered and bred through the Animal Science Department at University of Wisconsin-Madison^43^. WMS were approximately one year old at the start of treatment and had weights ranging from 70 to 100 kg. No inclusion/exclusion criteria was applied, so described results include analysis of all subjects. WMS were anesthetized throughout the course of imaging and treatment. All details regarding animal care and drugs administered can be found in the supplementary material. The animal procedures were approved by the University of Wisconsin Institutional Animal Care and Use Committee (IACUC). The drugs and methods of anesthesia and euthanasia were approved in compliance with American Veterinary Medical Association (AVMA) guidelines for anesthesia and euthanasia of swine. The committee review process ensured that all procedures were in compliance with ARRIVE guidelines. All experiments were performed in accordance with relevant guidelines and regulations.

The studies performed in WMS groups A and B differed in experimental design and protocol. The study of group A WMS focused on feasibility of validating pulmonary CT biomarkers with histopathology. In performing these initial experiments, study weaknesses were identified and addressed by developing improved methodology. The improved protocol was then used for a second round of experiments, performed in group B WMS. Methodology differences between the groups are highlighted throughout the following section and include improved dose distribution placement, improved spatial localization of regions of interest (ROI), and expansion of both the number of ROIs and histopathology performed in each ROI.

### CT Imaging

All ten WMS received CT imaging both pre- and three months post-RT. During CT imaging, WMS were sedated to significantly reduce motion artifacts and mechanically ventilated to control subjects’ breathing patterns. At each time point, subjects received three types of scans: maximum inspiration breath-hold (MIBH), four-dimensional CT (4DCT), and dynamic contrast-enhanced perfusion scans. The pre-RT MIBH scan was used for treatment planning and tracking distances to pathology points of interest. Pre- and post-RT 4DCTs and contrast scans were used to derive ventilation and perfusion information, respectively. During 4DCT acquisition, subjects were mechanically ventilated to a consistent tidal volume of 1 liter (L) and respiratory rate of 15 breaths per minute (BPM), matching the average tidal volume and respiratory rate of human subjects, and dynamic contrast CTs were acquired with the subject in a max inspiratory breath hold. Breath-hold maneuvers were achieved by setting the mechanical ventilator to maintain constant pressure. 4DCT acquisition and reconstruction methodology are detailed in Wallat *et al.*^17^. The dynamic contrast scan procedure is described in Wuschner *et al.*^19^.

### Radiation treatment delivery

All ten subjects underwent a five-fraction stereotactic body RT (SBRT) course of 12 Gy per fraction, totaling 60 Gy. Treatment planning and dose calculation were performed on the pre-RT MIBH CT. Treatment fractions were delivered following a standard clinical SBRT schedule, with one day between each fraction during weekdays and two days over the weekend. Subjects were mechanically ventilated to eight BPM during treatments with inverted breathing to hold inhale longer than exhale. While each group received the same total dose and fractionation scheme, the two groups of pigs were treated at different anatomical locations with different treatment machines. Treatment differences between the groups described by Wuschner *et al.*^44^ are summarized as follows.

**Figure 1:**
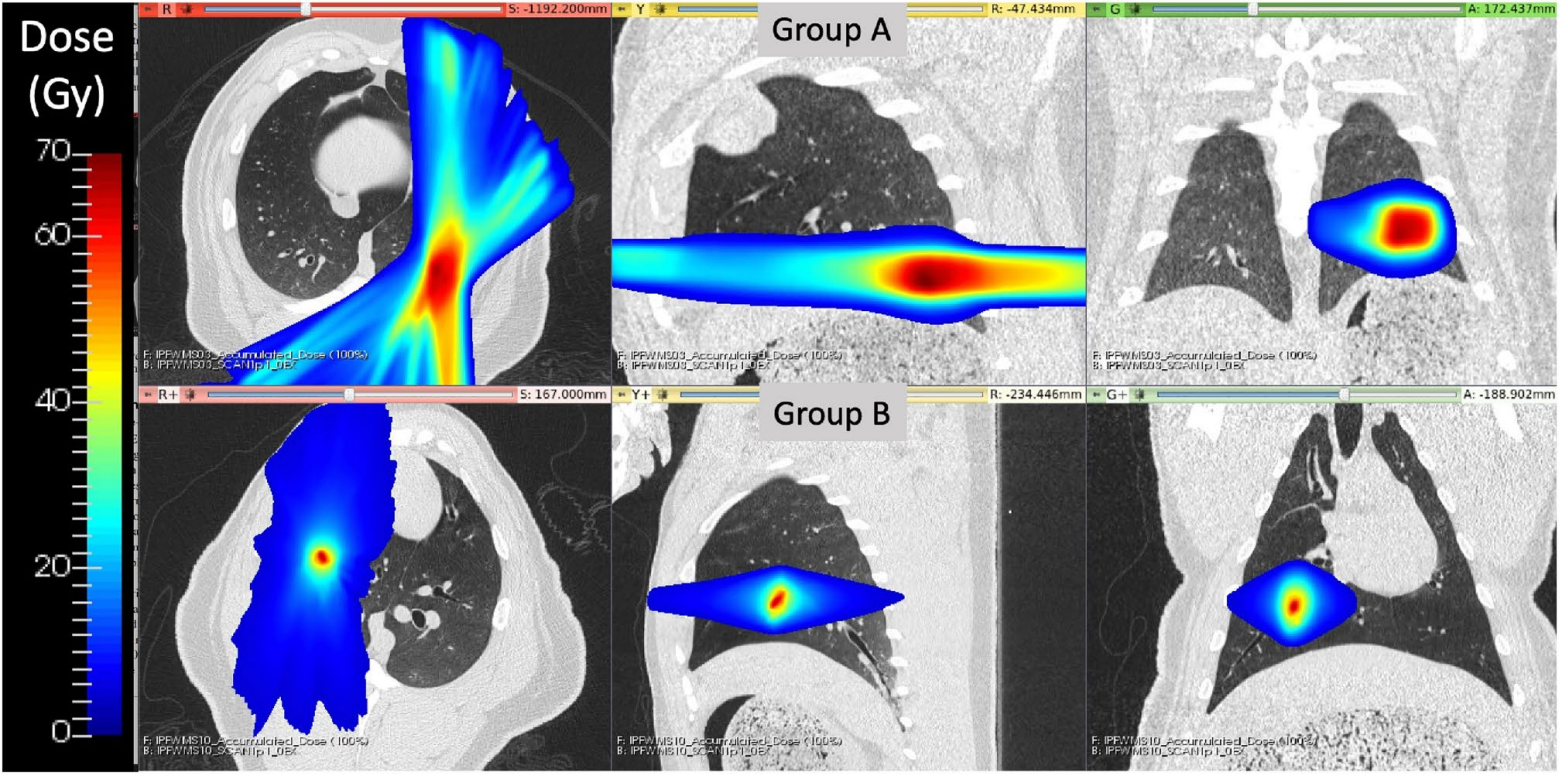
Delivered radiation dose distributions for WMS groups A and B show treatment differences. Group B had a more cranial dose distribution with a smaller region of targeted high-dose values. Reprinted from Wuschner *et al*.^44^ with permission.

Treatment commonalities between the groups included limiting contralateral lung dose (max dose point < 5 Gy) and the use of image-guided linear accelerators (LINACs) to maximize dose conformity and reduce the uncertainty of dose delivery due to respiratory motion. For group A WMS, the treatment planning target volume (PTV) was a vessel bifurcation in the lower left lobe. Treatments were delivered using an MRI-guided LINAC system (ViewRay, Cleveland, Ohio). For group B WMS, the PTV was centered on a vessel and airway in the subject’s right lung.Treatments were delivered on the Radixact^®^LINAC with motion Synchrony^47^ treatment system (Accuray Incorporated, Sunnyvale, CA). Figure 1 shows representative dose distributions delivered to subjects in each group.

### Imaging biomarker techniques

Pulmonary function biomarkers were derived from acquired CT scans using established techniques estimating ventilation (from 4DCT scans) and perfusion (from dynamic contrast scans) separately. Comparisons between these metrics were performed using two-tailed, paired student’s t-tests.

#### Perfusion metrics

**Figure 2:**
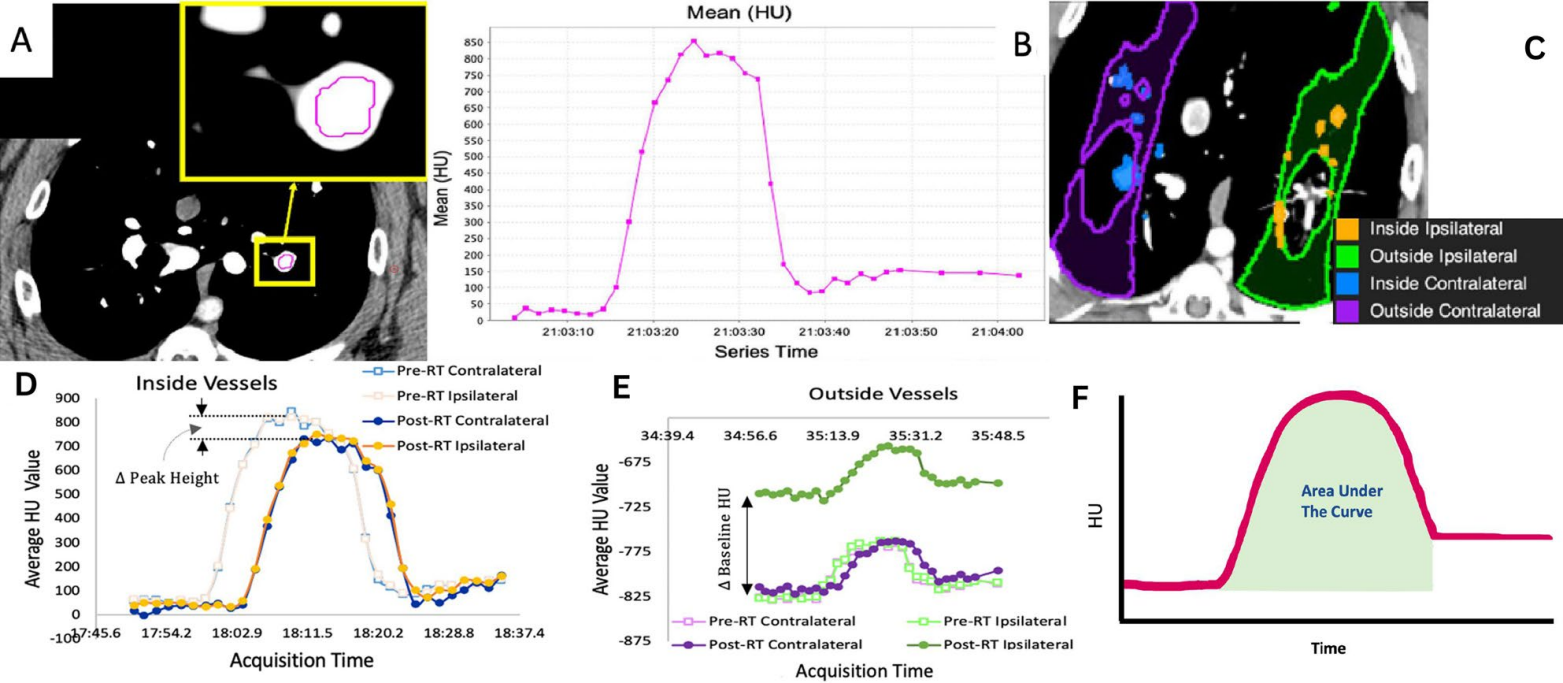
Summary of the perfusion imaging-based measurements. (**A**) Axial image from one frame of the dynamic contrast-enhanced CT showing an ROI placed in a vessel. (**B**) Mean Hounsfield Units (HU) value in the ROI from (**A**) across different frames of the dynamic CT. Each graph point represents a separate image acquisition. (**C**) Example of the contours used for vessel and non-vessel lung parenchyma dose binned measurements. (**D**) Measurement of peak HU change inside vessels. (**E**) Measurement of baseline HU outside vessels. (**F**) Area under the curve measurement to assess total contrast in the vessel. Images were adapted from our previous works^18,19^ and are modified and reprinted with permission.

Dynamic contrast CT scans was used to assess perfusion change. Details of the procedure and the numerous benefits of dynamic contrast CT over other contrast-based perfusion techniques have been thoroughly described in previous work^18,19^. To summarize, the dynamic contrast CT consisted of repeatedly scanning a region of interest (ROI) as contrast flows into and out of the lung. Throughout this process the subjects were placed in breath hold at a fixed tidal volume. By placing ROIs in different vessels, individual contrast-flow curves were produced (shown in panels A and B of Fig. 2). The flow of blood and contrast through each vessel was assessed from these contrast curves.

Multiple perfusion measurements were analyzed from the produced contrast curve. The first measurements quantified changes in peak Hounsfield Units (HU) inside vasculature from pre- to post-RT (Fig. 2, panel D) and the corresponding changes in baseline HU (prior to contrast injection) in the surrounding non-vessel lung parenchyma (Fig. 2, panel E). These measurements were performed in all subjects in vessels and surrounding parenchyma receiving a specific dose across multiple 10 Gy dose bins covering 0–60 Gy. This procedure was also performed in mirrored contours in the contralateral lung with contours shown in panel C of Fig. 2.

**Figure 3:**
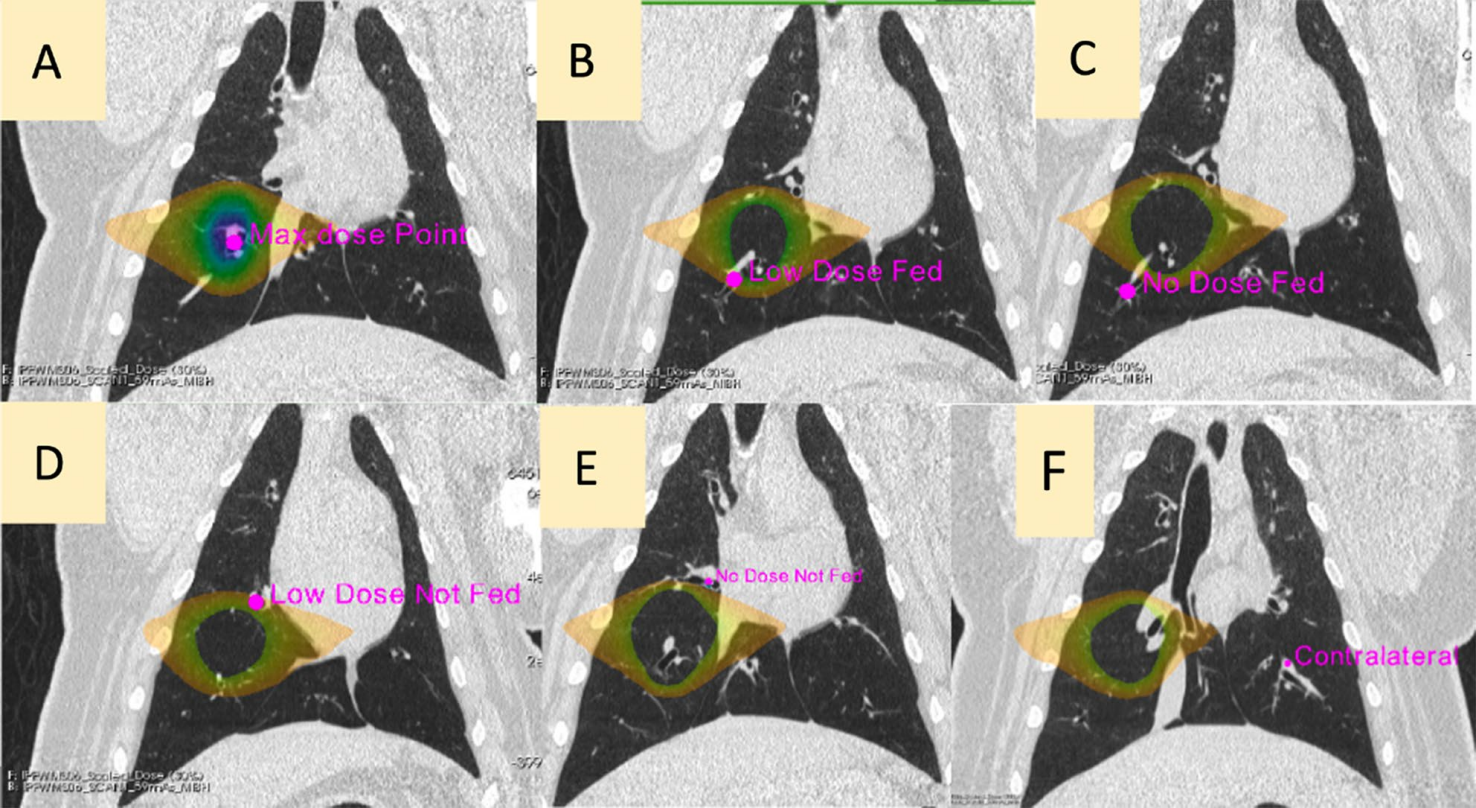
Analysis contours for the direct and indirect radiation response in a group B subject. Overlaid dose in panels B-F show delivered dose that classifies as low (5–20 Gy) or no (< 5 Gy) dose. Reprinted from Wuschner *et al.*^18^ with permission.

The second measurement, evaluated only in group B pigs, assessed the overall changes in total contrast flowing through different vessels as measured by the area under the contrast curves for vessels meeting different criteria. Figure 2, Panel F, shows an example of the area under the curve measurement. Figure 3 shows and describes the six different vessel locations where the total contrast area under the curve was analyzed for all group B pigs. The vessels include regions of the ipsilateral lung receiving high, low, and no doses, and a comparative contralateral lung vessel. Low and no-dose regions were separated into regions that were and were not fed by an irradiated vessel to understand indirect perfusion radiation response as previously highlighted^18^. All measurements utilized B-Spline image registration^48^ to allow for voxel-wise comparisons pre- to post-RT, and contours were placed using MIM software (MIM Software, Inc., Cleveland, OH). The specific methodology for performing each measurement is described in^18,19^.

#### Ventilation metrics

Local ventilation was derived from 4DCT scans using a Jacobian determinant (J) method previously developed by Shao *et al.*^14^ and referred to as LER-N. LER-N represents local ventilation by computing the local expansion ratio (LER) between different breathing phases and the end-exhale breathing phase. This method is unique because it uses multiple (N) 4DCT breathing phases instead of a single inhale/exhale pair. Consequently, LER-N better measures lung tissue expansion for regions expanding out-of-phase from the majority of lung. In the LER-N calculation, 4DCT breathing phases are registered to the end exhale breathing phase using a B-spline deformable image registration^48,49^. The Jacobian determinant of each image registration is calculated and combined to generate a voxel-level ventilation map estimating tissue elasticity. This estimated ventilation value represents the lung tissue expansion between minimal and maximal expansion points within the breathing cycle. The LER-N technique accounts for increased expansion that may occur outside the global minimal/maximal inhale volumes. Using 4DCTs to estimate ventilation also allows tidal volume (TV)-based effort correction, as previously explained by Wallat *et al.*^29^. This technique shows regional heterogeneity of lung ventilation and has primarily been validated through comparison to other imaging techniques, placing first in the 2019 American Association of Physicists in Medicine (AAPM) Grand Challenge: CT Ventilation Imaging Evaluation (CTVIE19). Two ventilation metrics were used for comparison with histopathology analysis point: the absolute ventilation value and the change in ventilation from before to after treatment.

4DCT-ventilation LER-N values typically ranged from 1 to 1.6, with higher values corresponding to more ventilation. Unfortunately, there is no consistently agreed upon threshold for defining highly ventilated lung. Flakus *et al.*^50^ identified and used a threshold of LER-N = 1.18 (≈ 18% voxel-level expansion) to define highly ventilated lung when predicting post-RT toxicity in humans. In this work, ventilation values in regions of interest were classified as ’High’ (LER-N≥ 1*.*2) or ’Low’ (LER-N≤ 1*.*1). Moderate ventilation values (1*.*1 <LER-N< 1*.*2) are challenging to interpret without further analysis of high-ventilating lung definitions.

Previous work defined radiation-induced damage to the lung based on the ratio of pre- to post-RT LER-N ventilation values (LER-N_*post*_*/*LER-N_*pre*_); the threshold for considering the lung to be damaged (i.e., a reduction in ventilation) was LER-N_*post*_*/*LER-N_*pre*_ < 0*.*94, corresponding to a ventilation reduction of at least 6%^29^. This selected threshold was based on biomarker repeatability in human subjects. While non-human subjects have previously demonstrated increased repeatability^45,51–53^, the threshold of 6% is used here as a conservative approach. A decrease in LER-N such that LER-N_*post*_*/*LER-N_*pre*_ < 0*.*94 is therefore considered ventilation decline when evaluated in this work. Similarly, LERN_*post*_*/*LER-N_*pre*_ > 1*.*06 is considered ventilation increase.

### Pathology points of interest

**Figure 4:**
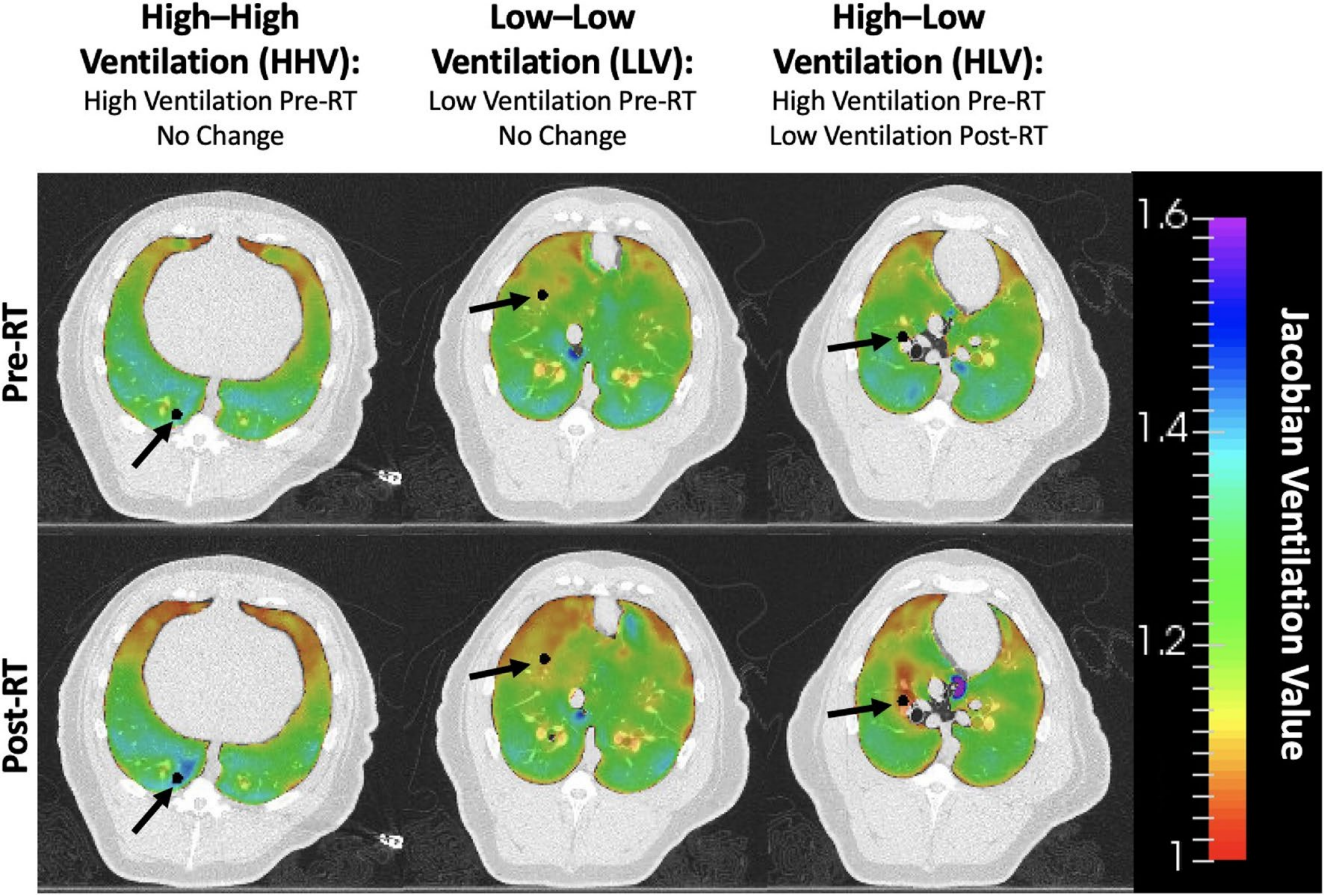
Three ventilation points of interest are shown, including high (HHV) and low (HLV and LLV) ventilation regions. HHV and LLV showed no ventilation change pre- to post-RT but HLV showed ventilation decline following treatment.

Post-mortem histopathology was performed on all pigs for multiple points of interest. The purpose of histopathology was two-fold: to understand subjects’ radiation response and to compare with ventilation and perfusion imaging biomarker metrics. For radiation response, direct and indirect points of interest (shown in Fig. 3) were evaluated and compared to perfusion and ventilation metrics. Additional ventilation points were evaluated to validate corresponding biomarkers. Points of interest were identified on CT images and mapped to post-mortem lungs (described in the measurement section). Points of interest are shown in Figs. 3 and 4 and described in Table 1 with differences between analysis points of groups A and B noted. Following analysis of group A subjects, the experimental design was improved to allow more extensive analysis in group B subjects. Increased analysis in group B includes more pulmonary function regions of interest and more histopathology observations.

**Table 1:** Description of Contours Analyzed on Each Subject. Contours only analyzed in the group B pigs are denoted with a *. Adapted from Wuschner et al.^18^ and reprinted with permission.

Contour Name	Description
Max Dose (MD)	The vessel contained in the PTV
Low Dose Fed* (LDF*)	A vessel in the ipsilateral lung receiving between 5 and 20 Gy that branches downstream of the vessel irradiated to the max dose
No Dose Fed* (NDF*)	A vessel in the ipsilateral lung receiving less than 5 Gy that branches downstream of the vessel irradiated to the max dose
Low Dose Not Fed* (LDNF*)	A vessel in the ipsilateral lung receiving between 5 and 20 Gy that does not branch from the vessel irradiated to the max dose
No Dose Not Fed* (NDNF*)	A vessel in the ipsilateral lung receiving less than 5 Gy that does not branch from the vessel irradiated to the max dose
Contralateral (CON)	A vessel in the contralateral lung (received no dose) at the approximate mirrored location as the point of max dose in the ipsilateral lung
High Ventilation* (HHV*)	A parenchymal point in the ipsilateral lung that ventilation biomarkers designated as highly ventilated both pre- and post-RT
Low Ventilation* (LLV*)	A parenchymal point in the ipsilateral lung that ventilation biomarkers designated as having low ventilation both pre- and post-RT
High to Low Ventilation* (HLV*)	A parenchymal point in the ipsilateral lung that ventilation biomarkers designated as high ventilation pre-RT but low ventilation post-RT

#### Direct radiation response analysis points

Direct radiation response was measured by evaluating the point of maximum dose (MD) in all ten WMS (Fig. 3, Panel A). The MD point was centered in the parenchyma for group A and in a vessel for group B subjects due to differences in irradiation location (described in the previous section). The MD point was identified by overlaying the delivered dose distribution on the treatment planning scan (pre-RT MIBH). A control point in the contralateral lung (CON) was used as a comparison benchmark in both WMS groups. The CON point was localized to approximately the same spot as the MD point in the ipsilateral lung (Fig. 3, Panel E) and did not receive any dose (< 5 Gy).

#### Indirect radiation response analysis points

For group B WMS, the indirect radiation response was measured in addition to the direct radiation response. Four indirect radiation response points of interest were evaluated in vessel-centered sections with surrounding parenchyma to compare to perfusion and ventilation biomarker metrics. The points were classified by both dose level and whether or not they were fed by an irradiated vessel. For dose levels, ‘Low Dose’ was defined as receiving 5-20 Gy and ‘No Dose’ was defined as receiving < 5 Gy. Vessel points that branched from the vessel irradiated with the maximum dose were considered ‘Fed’ and those that did not were considered ‘Not Fed’. Therefore, the four indirect points of interest were Low Dose Not Fed (LDNF), Low Dose Fed (LDF), No Dose Not Fed (NDNF) and No Dose Fed (NDF). Table 1 lists all radiation response points of interest, including these indirect points for group B subjects. All indirect response points of interest were first identified using the pre-RT dynamic perfusion CT and the dose distribution in MIM, then registered to the pre-RT MIBH scan for anatomical measurements (discussed later). Contours were then deformably propagated to each frame of the dynamic perfusion CT in MIM and transferred to the previously registered post-RT scan.

#### Additional ventilation analysis points

The group B pigs also had additional ventilation-based points of interest identified for analysis based on ventilation maps generated from 4DCT scans. These tissue sections were centered in the parenchyma. Once generated, pre- and post-RT ventilation maps were registered to the treatment planning MIBH CT using B-spline deformable image registration^48^. Registered ventilation maps were used to select three ventilation-based points of interest for each subject, classified by their pre- and post-RT ventilation values: a high-high ventilation (HHV) point, a low-low ventilation (LLV) point, and a high-low ventilation (HLV) point. All three ventilation points were placed within the ipsilateral lung. The HHV point was placed in a lung region with high ventilation values both pre- and post-RT. The HLV point had high ventilation pre-RT and decreased to low ventilation post-RT, whereas the LLV point had low ventilation at both the pre-RT and post-RT time points. All three points are shown for one subject in Fig. 4 and described in Table 1. All three ventilation-based points were chosen to be easily accessible by following airways to increase location accuracy for performing pathology. Additionally, each selected point was within a region of similar ventilation values with the same high or low classification.

### Anatomical measurements for pathology

Post-mortem histopathological analysis was performed on a section of lung taken from each of the analysis points described in Table 1. Following euthanasia, the lungs were removed from the subject and immediately re-inflated and filled using a formalin solution. A catheter was placed down the main bronchi and a syringe was used to push formalin through to fully expand all lobes to their in-vivo max-inspiration inflation level as much as possible. Once the desired inflation was achieved, the main bronchi were tied to prevent leakage and the lungs were placed in a bath of formalin such that they could float and avoid deformation prior to trimming. For group A subjects, necropsy was performed within a month following the 3-month post-RT CT imaging. Lungs were preserved for roughly one year before histopathology could be performed. Group B subjects experienced an expedited timeline; necropsy was performed within a few days of 3-month post-RT imaging and lungs were preserved for approximately six months prior to histopathology analysis.

#### Group A WMS

For group A subjects, localization of the MD point was performed using measurements from the CT images in MIM software. However, it should be noted that while best efforts were employed to fully fix the lungs, they were stored for a prolonged period before trimming, and there was some contraction of the tissue, causing a discrepancy between the measurements made on imaging and observed in the lungs. All possible efforts were made to correlate landmarks on imaging with extracted lung landmarks to localize the correct anatomical MD and CON points. Since the dose was targeted to a parenchymal spot in the lung and not necessarily close to a large major structure, spatial accuracy is limited and point-of-interest sections could be slightly misplaced.

#### Group B WMS

For group B subjects, PTV placement allowed for improved methods of identifying anatomical locations. Localization of points of interest for tissue trimming and eventual histopathology analysis was achieved by following airways from the carina to each point. All radiation response analysis points were placed in a vessel adjacent to an airway and additional ventilation points were placed in the parenchyma just outside an airway.

Beginning at the carina, fiducial points were used to mark airway branch points leading to the points of interest using 3D slicer version 4.5.0-1 (www.slicer.org^54^). All points were identified on a pre-RT MIBH CT scan used for treatment planning. The Euclidean distance between fiducial markers was used to map the distance traveled along airways to reach the points of interest. When trimming the tissue, these distances were followed to reach each point, and the points were marked with ink. An example of applying slicer-based fiducial marking measurements to post-mortem lungs is shown in the supplemental material. Since the lungs were well-inflated and the airways were used for localization (a structure that does not contract significantly post-necropsy), the measurements on imaging between branches were found to match well with the measurements observed in the subjects. This methodology was tested and validated prior to use by taking a pig from another study, injecting a small amount of ink into the lung prior to removal of the lungs from the body cavity, and attempting to locate the dye from imaging measurements. The attempt was successful and we estimate this method is spatially accurate to within 2-3 mm.

Following measurements and tissue dyeing with ink, tissue was sectioned for histopathology analysis. At each point of interest, an experienced pathologist cut approximately 1” square sections surrounding these marked points to create a cassette for each sample containing vessel, airway and surrounding lung parenchyma.

### Pathology analysis

Each trimmed section was analyzed microscopically with Masson’s Trichrome (all pigs) and Hematoxylin and Eosin (H&E) stains (group B pigs only) applied. Stained sections were analyzed by an experienced pathologist to identify evidence of tissue damage and/or structural changes.

#### Trichrome Stain

The purpose of the trichrome stain is primarily to identify collagen and muscles. Conventional trichrome staining techniques were used in which a red dye in dilute acetic acid was applied first to overstain all components. Next, polyacid was applied to remove the red dye from collagen and some other components by displacement. Finally, a second acid dye (blue) in dilute acetic acid was applied, which displaces the polyacid and causes the collagen to be stained blue. The result shows collagen stained blue, nuclei stained brown, muscle tissue stained red, and cytoplasm stained pink^55^.

For group A subjects, collagen presence was noted as a binary finding (collagen present or not). For group B subjects, the amount of collagen at all nine points of interest was classified by a scale with the following categories: no significant, minimal, mild, moderate, marked and severe collagen deposited beyond what is part of normal anatomy. If the observed collagen level fell between two categories, it was listed as ranging between both, i.e., minimal-mild.

#### H&E stain

In the H&E stain, the Hematoxylin colors chromatin and mucin blue and nuclei purple-blue, and the Eosin colors cytoplasmic structures and the extracellular matix varying shades of red to pink^55^. This stain allows identification of structural changes and histologic diagnoses. In group A pigs, structural changes were noted, but in group B, specific histologic diagnoses were made. These diagnoses include fibrosis, inflammation, necrosis, vascular proliferation and many more. These diagnostic results were quantified based on how many WMS (out of the five in group B) they were observed in.

## Results

### Imaging biomarker findings

#### Perfusion

Direct and indirect radiation response of perfusion biomarker metrics have been previously reported and key figures are given in the supplementary material. The direct radiation response points with perfusion measurements assessed in both the group A and B pigs showed a reduction in HU value inside the vasculature and an increase in HU outside the vessels post-RT. This is illustrated in the contrast curves shown in panels D and E of Fig. 2 and was previously reported in Wuschner *et al.*^19^. Furthermore, the reductions in HU inside the vessels were strongly correlated with increases in the lung parenchyma surrounding those vessels, which is shown in the supplementary material. The severity of the reduction was dose dependent and the strong correlation was valid for dose bins above 25 Gy. The hypothesized interpretation of these results reported in Wuschner *et al.*^19^ was that this correlation of measurements indicated leakage of blood from the vessel to the surrounding lung parenchyma above a certain dose threshold.

Indirect perfusion measurements assessed in the group B pigs showed that perfusion was reduced regardless of delivered dose for regions fed by highly irradiated vasculature. This observation was quantified by the change in area under the contrast curve measurements shown in the supplementary material. The area under the curve was statistically significantly reduced (*p* < 0.05) for the max dose and fed contours but not in the not fed or contralateral contours. The reduction in area under the curve indicates an overall reduction in contrast imaged in the vessels and was reported in Wuschner *et al.*^18^. In this work, these results were hypothesized to indicate reduced perfusion due to vascular damage preventing the flow of contrast. However, the previous work was unable to speculate mechanism.

There was one pig for which the “No Dose Fed” contour could not be analyzed due to a mistake in image acquisition. This was due to the incorrect selection of the appropriate field of view for the dynamic contrast scan, and thus there was not enough overlapping anatomy imaged pre- and post-RT to create a contour meeting that classification. All other contours were able to be analyzed on this subject.

#### Ventilation

No subject showed ventilation increase (LER-N_*post*_*/*LER-N_*pre*_ > 1*.*06) in any region of interest. The direct radiation response of ventilation biomarkers (at MD point) in all ten pigs showed lower ventilation values post-RT than pre-RT. All five group A WMS and four out of five group B WMS showed ventilation decline (LER-N_*post*_*/*LER-N_*pre*_ < 0*.*94). The remaining group B WMS had LER-N_*post*_*/*LER-N_*pre*_ = 0*.*95 at the MD point. Ventilation decline was not observed in any of the subjects at the CON point. Eight of ten subjects had a low ventilation value (LER-N_*post*_ ≤ 1*.*1) at the MD point and only one was classified as low ventilation in the contralateral lung (CON point). These results demonstrate ventilation decline after treatment in high-dose regions of the ipsilateral lung but not in the unirradiated areas of the contralateral lung. Therefore, CT-ventilation biomarkers demonstrate direct radiation damage via a reduction in ventilation.

**Figure 5:**
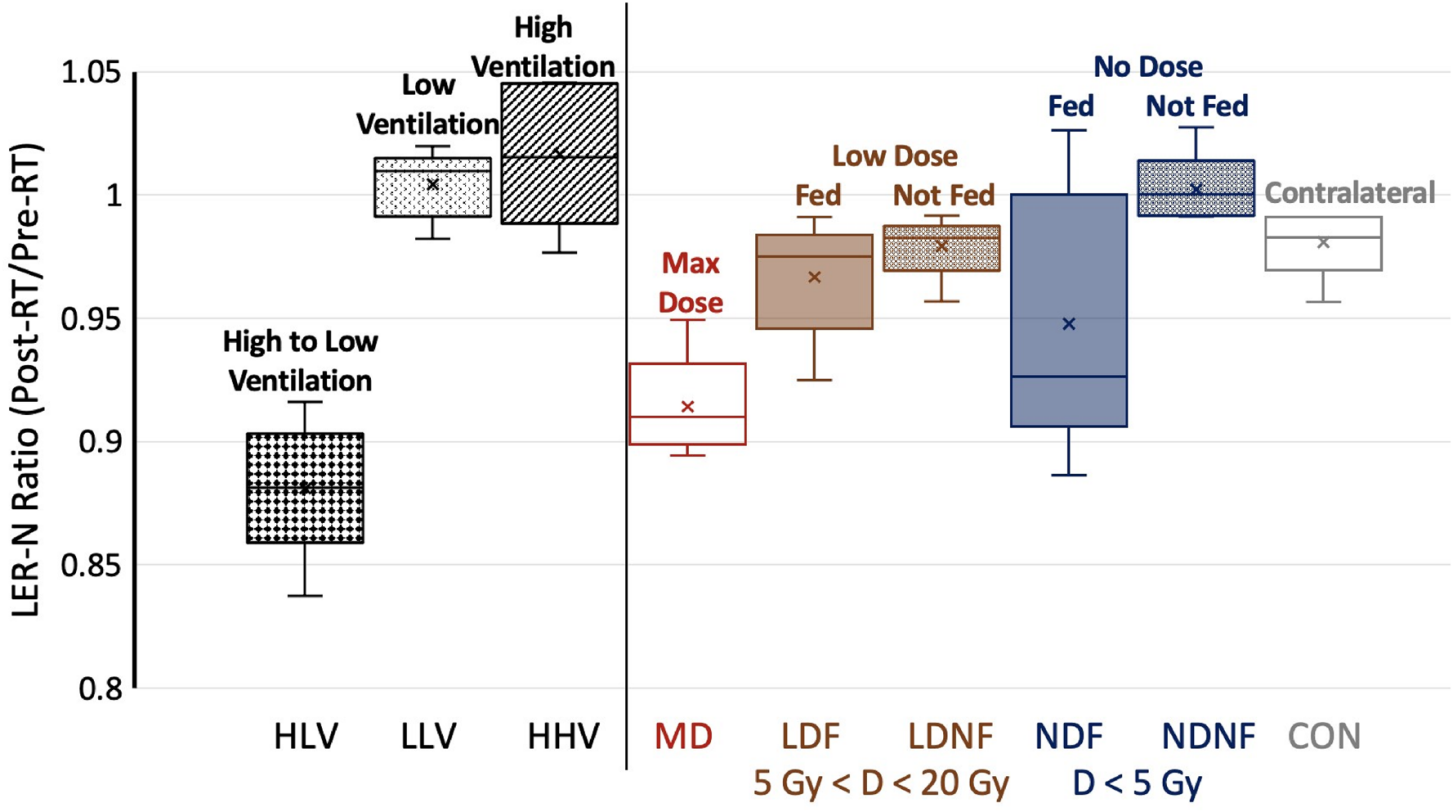
Ventilation change (LER-N_*post*_*/*LER-N_*pre*_) at all nine points of interest in group B WMS. LER-N_*post*_*/*LER-N_*pre*_ = 1 indicates no change, and LER-N_*post*_*/*LER-N_*pre*_ < 0*.*94 was defined as ventilation decline. The reduced ventilation is most evident at HLV and MD points. Fed points show more ventilation decline than not fed points but are not significantly different (*p* > 0*.*12). Acronyms: HLV = High–Low Ventilation, LLV = Low–Low Ventilation, HHV = High–High Ventilation, MD = Maximum Dose, LDF = Low Dose Fed, LDNF = Low Dose Not Fed, NDF = No Dose Fed, NDNF = No Dose Not Fed, CON = Contralateral Control.

Group B WMS had seven additional points of interest evaluated in addition to the direct radiation damage and contralateral control points. These included four points evaluating indirect radiation damage and three selected based on their ventilation values. Changes in ventilation from pre- to post-RT, as quantified by the LER-N ratio, are shown for all nine points of interest from Table 1 for the five group B subjects in Fig. 5. Xs within the box-and-whisker plots denote the mean value for each contour. The left panel shows black patterned boxplots for ventilation-based points of interest (HLV, LLV and HHV as defined in Fig. 4).

HHV points had high ventilation values ranging from 1.25 to 1.48 and did not show pre- to post-RT ventilation decline, with a mean and standard deviation Jacobian ratio values of LER-N_*post*_*/*LER-N_*pre*_ = 1*.*02±0*.*03. LLV points also did not show any post-treatment ventilation decline (LER-N_*post*_*/*LER-N_*pre*_ = 1*.*00±0*.*01) and had low ventilation values ranging from 1.02-1.1. HLV points ranged from 1.2–1.27 (pre-RT) to 1.03–1.1 (post-RT), all showing ventilation decline. On average, HLV had LER-N_*post*_*/*LER-N_*pre*_ = 0*.*88±0*.*03, corresponding to a 12% ventilation decline. The HLV post- to pre-RT LER-N ratio was significantly different than that of the HHV (*p* = 0*.*005) and LLV (*p* = 0*.*001) points.

The right panel shows all the vessel-centered radiation response points from Fig. 3. The max dose region had a significantly different LER-N ratio than the contralateral lung (0.91 vs. 0.98, *p* = 0*.*003). At indirect points of interest, ventilation values were moderate-high pre-RT (range 1.1–1.35) and low-high post-RT (range 1.09–1.25). For all pigs, there was no ventilation decline indicated at either of the ‘Not Fed’ points of interest (NDNF and LDNF). For the ‘Fed’ points, the results were more mixed across subjects; in the low dose region, one WMS (subject C) showed ventilation decline at the LDF point, while three WMS (subjects C–E) showed ventilation decline in the no dose region (NDF). LER-N ratios were not significantly different between fed and not fed contours in the low-dose (0.97 vs. 0.98, *p* = 0*.*35) or no-dose (0.94 vs. 1.00, *p* = 0*.*12) regions. Indirect radiation-induced damage points of interest are shown in the brown (low dose) and blue (no dose) box plots of Fig. 5. This plot visually demonstrates more ventilation decline in fed contours. This result agrees with previous work by Wallat *et al*.^17^ that saw a greater ventilation decline in regions fed by an irradiated airway, but the difference in ventilation was not statistically significant.

To summarize, ventilation biomarkers indicate pre- to post-RT ventilation decline at MD and HLV points and no decline in any subject at CON, HHV, LLV, NDNF, and LDNF points. Ventilation decline is seen in some, but not all, subjects at the LDF and NDF points.

### Histopathology findings

#### Group A pigs

**Figure 6:**
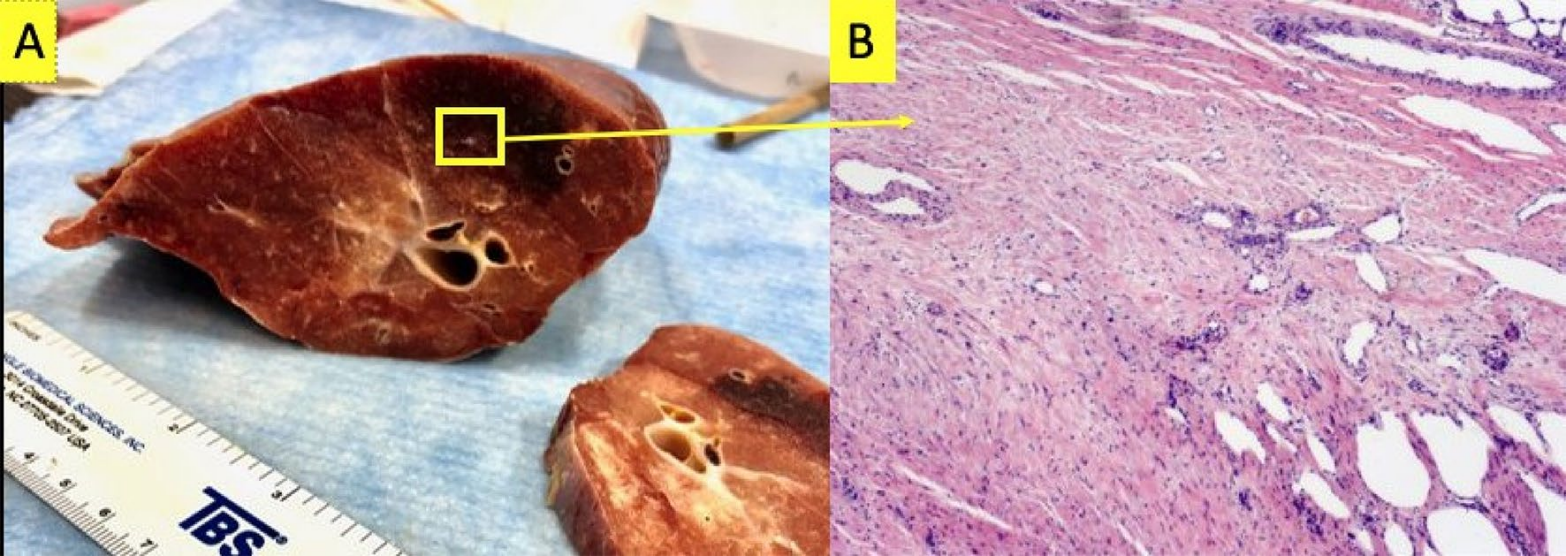
Primary pathology finding from group A 5 WMS study. (**A**) Image of a cross-section of the lung at the point of max dose. A yellow box is drawn to indicate where the sample was taken. The lung is discolored suggesting hemorrhaging is observed. (**B**) Enlarged image of the slide created from this sample’s H&E stain. The stain confirms the proliferation of blood vessels into the lung parenchyma in an area of fibrosis. This was observed in 4 out of 5 WMS.

**Figure 7:**
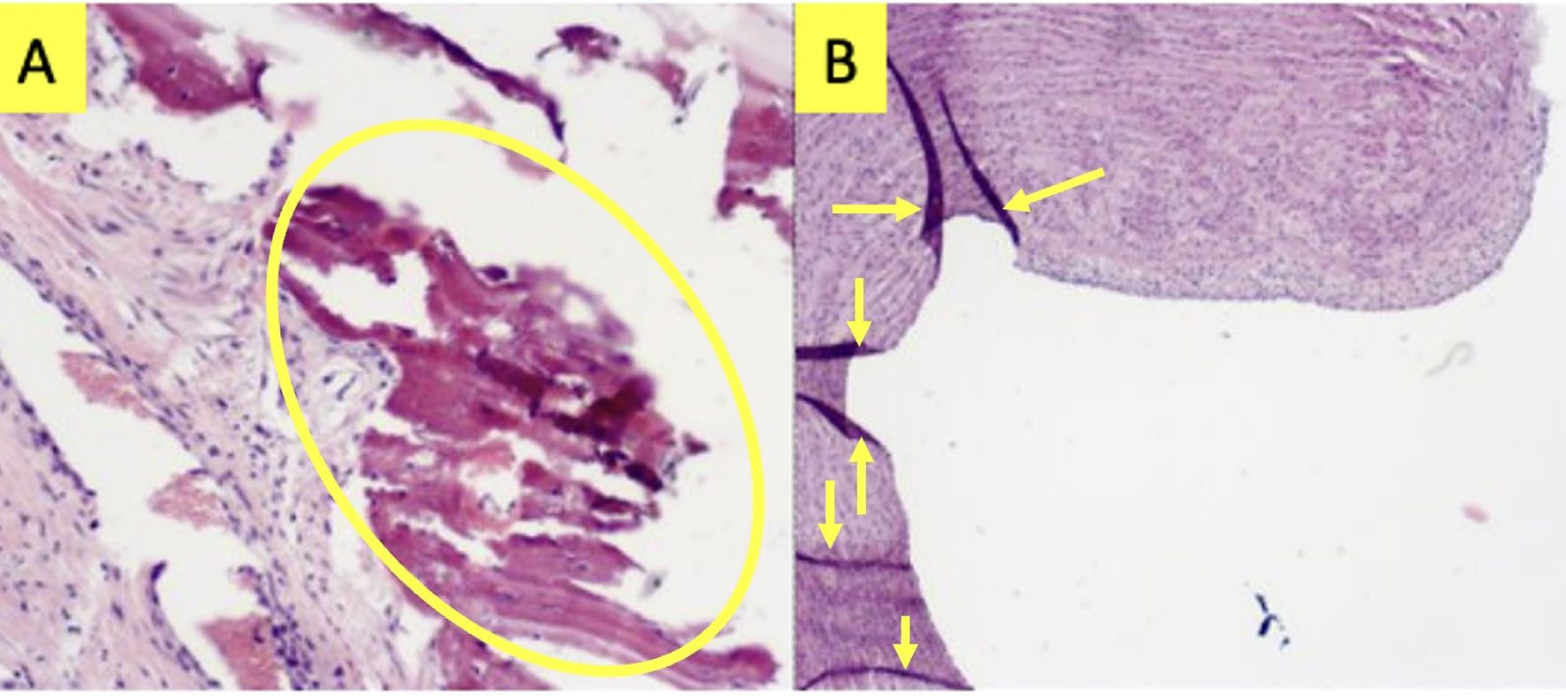
Images of slides post-trichrome staining indicating additional effects beyond the proliferation into the lung parenchyma. (**A**) Mineralization and potential meta-plastic bone (seen in 1 subject). (**B**) Fibro-collagenous proliferation between the internal elastic lamina and endothelial cell layer (seen in 3 subjects).

**Figure 8:**
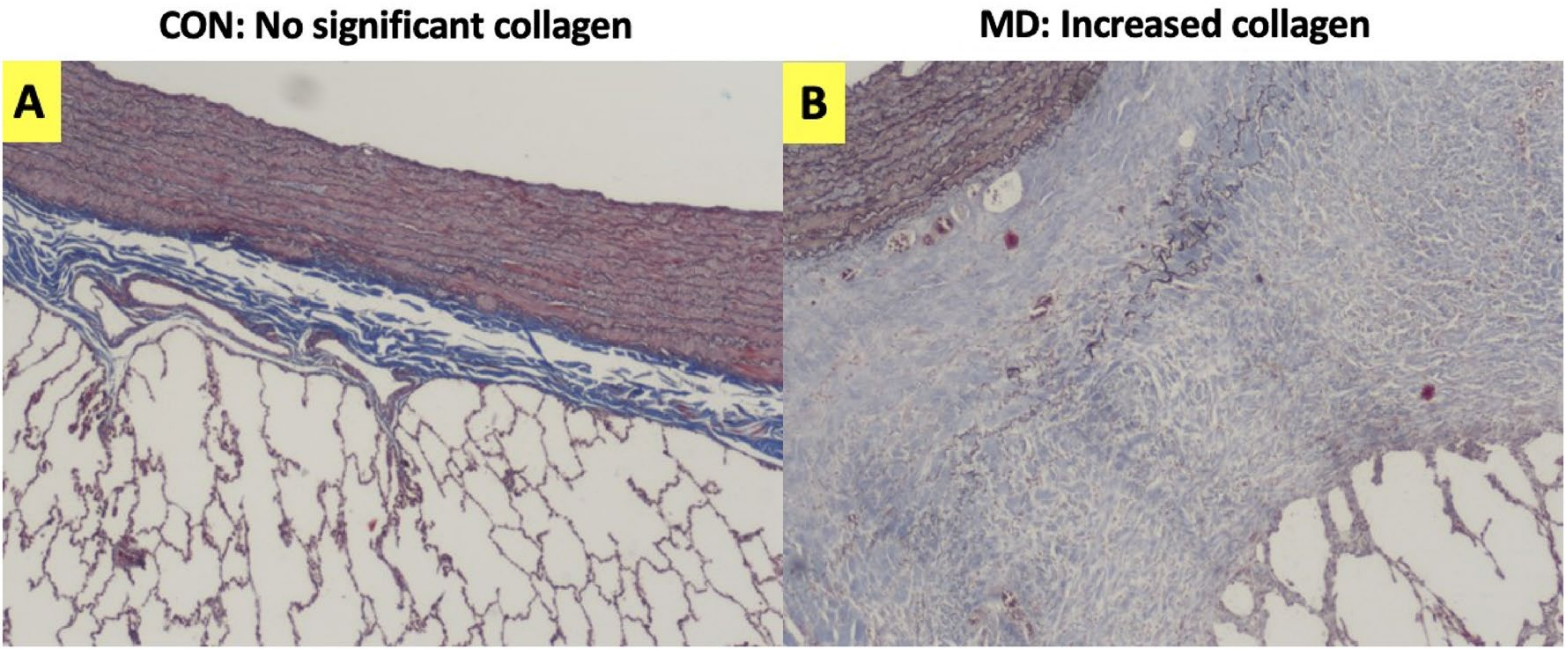
Increased collagen is seen with trichrome stain. (**A**) A sample from the contralateral lung section (CON) on the left is compared to (**B**) a sample from the maximum dose (MD) region.

The results of the pathology performed on the group A pigs to assess direct damage are summarized in Figs. 6 and 7. In all but one WMS, the proliferation of red blood cells into the lung parenchyma was observed (but not in the control slide). Additionally, other effects were observed in the ipsilateral lung such as fibro-collagenous proliferation (3 subjects), immune response (1 subject), and mineralization (1 subject). Overall, increased evidence of radiation-induced damage was seen at the MD point compared to CON, and this is demonstrated by collagen presence in Fig. 8.

#### Group B pig trichrome stain

**Figure 9:**
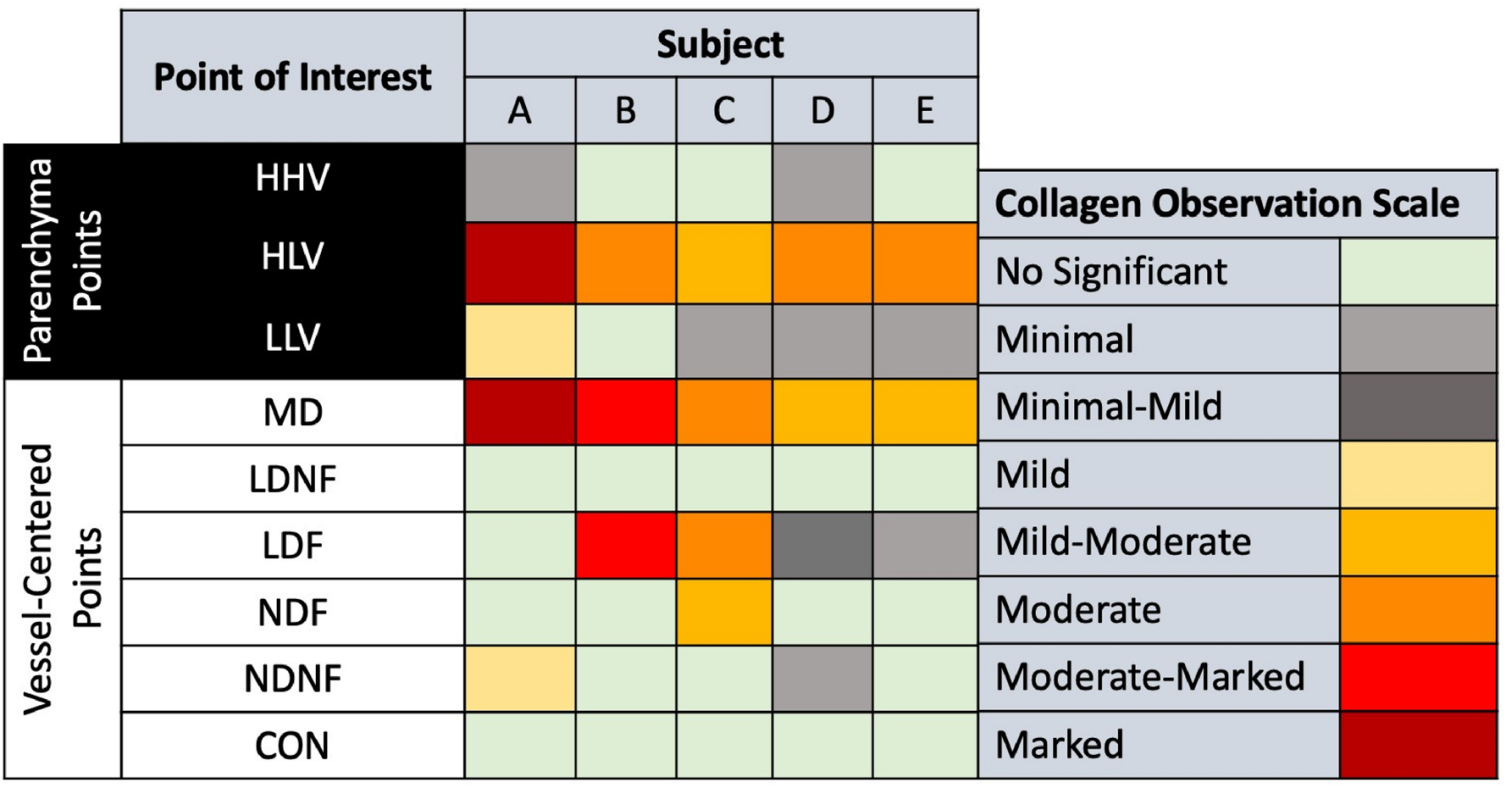
Degree of collagen deposition from group B pigs trichrome stains. The degree of collagen observed is shown for each contour in each pig (A-E) and is scored on a scale of no significant observation to marked collagen. Contours are labeled as high-high ventilation (HHV), high-low ventilation (HLV), low-low ventilation (LLV), max dose (MD), low dose fed (LDF), low dose not fed (LDNF), no dose fed (NDF), no dose not fed (NDNF), and contralateral (CON).

**Figure 10:**
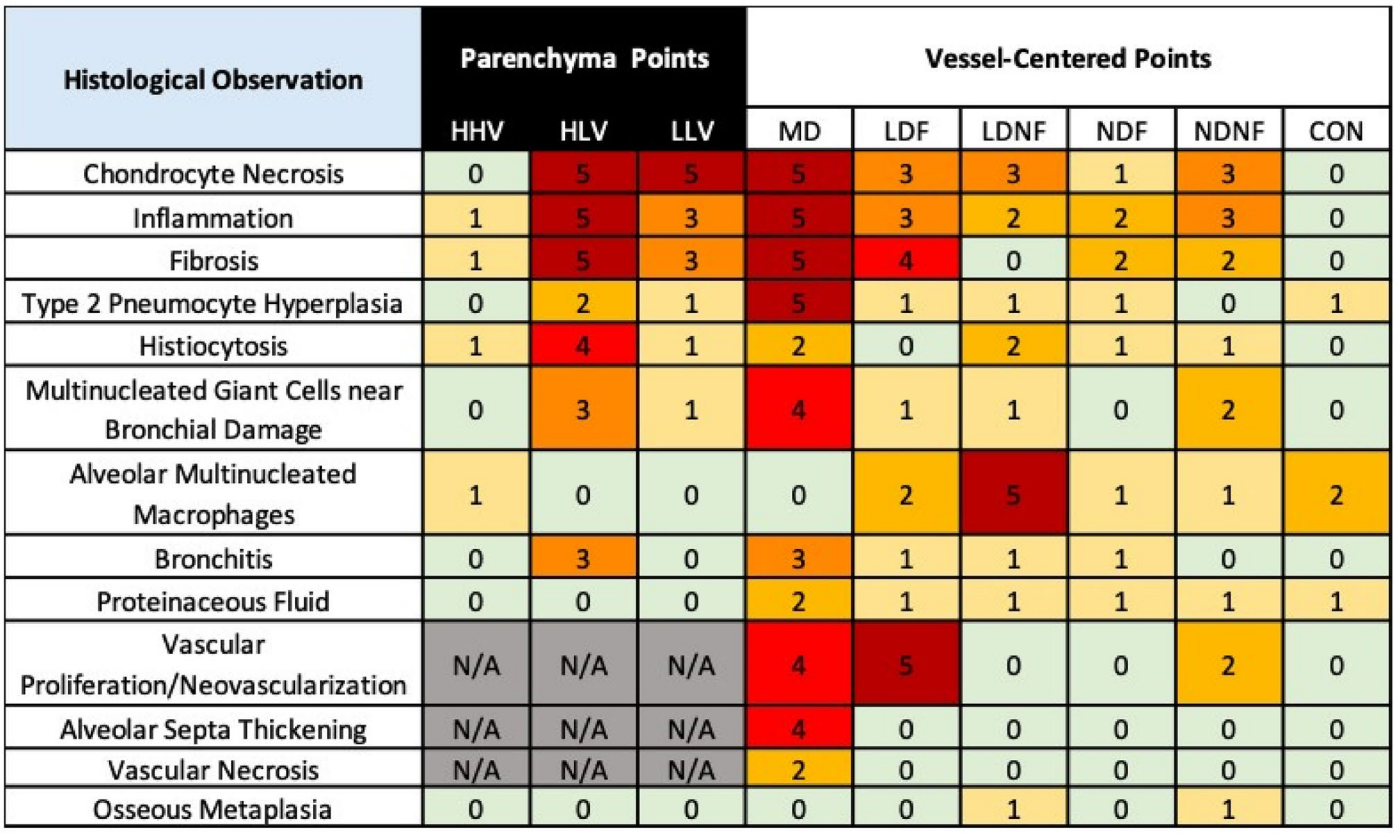
Summary of histological diagnoses at each analysis point in the group B pigs. Number in each cell corresponds to the number of subjects (out of a total of 5) that experienced each specific condition in each specific contour. Contours are labeled as high-high ventilation (HHV), high-low ventilation (HLV), low-low ventilation (LLV), max dose (MD), low dose fed (LDF), low dose not fed (LDNF), no dose fed (NDF), no dose not fed (NDNF), and contralateral (CON).

The pathology findings in the group B pigs are summarized in Figs. 9 and 10. Figure 9 summarizes the results of the trichrome stains applied. Observed collagen deposition varied from no significant observations to marked collagen. No subject showed severe collagen deposition (the highest degree of collagen on the evaluation scale) at any contour. However, there were general trends in the different contours analyzed.

In the ventilation contours, the high-high ventilation (HHV) contour showed very little collagen with 3 out of 5 subjects showing no significant collagen and 2 showing minimal. The two that showed minimal collagen were labeled as suspect fixation artifacts by the pathologist. The high-low ventilation (HLV) contour consistently showed collagen presence in all subjects ranging from mild-moderate in one, moderate in three, to marked in the fifth. Finally, the low-low ventilation (LLV) contour showed primarily minimal collagen in 3 subjects with one subject showing no significant collagen and one showing minimal-mild. HLV showed the most collagen, LLV the second most and HHV the least of all ventilation points.

The direct radiation response MD contour consistently showed collagen presence ranging from mild-moderate to moderate-marked. In comparison, no significant collagen was seen in any subject in the CON contour. For indirect points, not fed contours showed mild collagen presence at best, with LDNF and a majority of NDNF contours showing no significant collagen for any subject; NDNF had two subjects with noted collagen presence: one with minimal and one with mild. In contrast, the LDF contour showed a wide variety in collagen presence with each subject showing a different level ranging from no significant to moderate-marked. The NDF contour more consistently showed low levels of collagen with four showing no significant and one mild-moderate.

Overall, high-high ventilation, not fed and control regions showed very little collagen. Fed and low-low ventilation regions showed some collagen but were less consistent across subjects. The max dose and high-low ventilation regions showed the largest amount of collagen.

#### Group B pig histologic diagnoses

Similar to the group A results, there were some diagnoses in the group B subjects that were consistent across subjects, and several others that were less common and only were observed in a subset of subjects (defined as majority: 3 or more). The frequency of observation for each observed diagnosis is summarized in Fig. 10 for all analysis points. The number in each cell of the table gives the number of subjects (maximum of 5) that were reported to have the listed diagnosis in the specified contour. Strength of diagnoses (from minimal to marked) are described in the current section and in the discussion section comparing correlation between biomarkers and pathology.

No notable diagnoses were consistently observed in the HHV contour for the ventilation points. Observed diagnoses (in at most one subject) were all minimal in strength except for mild inflammation. The HLV contour had multiple consistent diagnoses with chondrocyte necrosis, inflammation, and fibrosis for all subjects, and four subjects reported histiocytosis. Additionally, multinucleated giant cells and bronchitis were observed in a subset of subjects. In two of the three subjects with bronchitis, it reached bronchial ulceration. The strength of these diagnoses was primarily moderate, with a few mild and marked diagnoses. The only consistent diagnosis in the LLV contour was chondrocyte necrosis, and three subjects were also diagnosed with inflammation and fibrosis. All other diagnoses were shown in one subject at most. LLV diagnoses were at most mild-moderate in strength. Of the ventilation points of interest, histologic diagnoses showed the most damage in HLV contours, second most in LLV, and least in HHV.

For direct radiation response contours, the MD point consistently presented with chondrocyte necrosis, inflammation, fibrosis, and type II pneumocyte hyperplasia across all subjects. Additionally, four subjects presented with alveolar septa thickening and multinucleated giant cells. A subset of subjects also experienced histiocytosis, bronchitis and/or vascular proliferation. In two of these subjects, the bronchitis got as bad as bronchial ulceration. The MD diagnoses ranged from mild to marked in strength. Comparatively, the CON contour did not have any histological diagnoses observed consistently.

For indirect radiation response contours, the LDF contour showed fibrosis in 4 subjects. Subsets of subjects experienced inflammation, chondrocyte necrosis, and neovascularization. Notably, the LDNF contour did not show any fibrosis, consistent with no collagen presence from the trichrome results of Fig. 9. The LDNF contour also consistently showed multinucleated macrophages, with a subset experiencing chondrocyte necrosis. Both the NDF and NDNF showed no diagnoses consistently, but inflammation and fibrosis in 2–3 subjects. The NDNF contour also showed chondrocyte necrosis in a subset as well as multinucleated giant cells and vascular proliferation in two subjects.

In summary, high-high ventilation and control points showed very minimal evidence of any diagnosis. No dose regions (both fed and not fed) did not consistently show any diagnoses across subjects, with each being observed in 0-3 pigs. Low-dose regions (both fed and not fed) show a few diagnoses across most subjects with increased damage. High-low ventilation and the max dose point consistently show multiple diagnoses.

## Discussion

Overall, histopathology showed several correlations with imaging biomarker analysis. In the perfusion analysis, the pathology observations from groups A and B pigs support the main imaging hypothesis of vascular radiation-induced damage leading to red blood cell leakage from the vasculature. In all but one pig, hemorrhaging with proliferation of red blood cells into the lung parenchyma was observed (but not in the control slide). Red blood cell leakage combined with observations of vascular damage support perfusion biomarker findings of a radiation-induced decline in perfusion. In the indirect analysis, pathology observations such as vascular proliferation and fibrosis also correlated with measured imaging changes in vasculature that were not directly irradiated but that were supplied by an irradiated vessel. These results are discussed in further detail in sections on perfusion radiation response.

Correlations were also found in the ventilation analysis. Regions where ventilation was reduced by treatment showed consistent evidence of lung damage (increased collagen and/or fibrosis) but consistently high ventilation regions did not. Consistently low ventilation regions were in between, with some damage noted. An over 90% agreement rate between biomarker metrics and histopathology findings provides strong evidence that Jacobian-based ventilation biomarkers are representative of underlying lung function. The specifics of this analysis are discussed in sections on ventilation radiation response.

### Interpretation of pathology observations

The diagnoses listed in Fig. 10 reflect the findings within the entire approximately 1" tissue section. Some observations and diagnoses are difficult to fully interpret due to post-mortem lung preservation methods. In particular, alveolar contents and pneumocyte characteristics are challenging to interpret, along with the exact antemortem locations of alveolar macrophages, multinucleated giant cells, and inflammatory cells, such as lymphocytes. Formalin infusion potentially displaced these features from their originating location.

The type 2 pneumocyte hyperplasia diagnosis is also questionable. This diagnosis was made based on the observation of plump reactive type 2 pneumocytes that lined small regions of the alveoli. However, histologically similar findings were noted in the contralateral lung of one subject. Pneumocytes are not often preserved with formalin infusion^56^, making it unclear if this was a radiation-induced effect. However, the repeatability of this diagnoses in max dose irradiated regions suggests that it may have been radiation-induced, especially since the diagnosis aligns with ventilation and perfusion imaging biomarker findings. The fixation method elected in this study was chosen to optimize microanatomical analysis of airway structure, inflammation, fibrosis, and vascular damage as relates to imaging. Alternate fixation methods could be used in future studies to elucidate the effects on pneumocytes.

Additional observations that are difficult to interpret are the accumulation of eosinophilic amorphous proteinaceous fluid in interstitial spaces and airways. In some cases, this can be interpreted as edema, but despite rapid collection and fixation of the lungs after euthanasia, there may be a contribution of an artifact of postmortem extravasation from vessels. Displacement of fluid from antemortem locations as a result of formalin infusion into airways and subsequent dispersal is also possible.

Despite these challenges, there are a couple diagnoses that we can confidently conclude were radiation-induced due to their presence in irradiated and damaged regions but not in other regions. These histologic features also are not expected to be significantly altered by chosen methods of fixation^56^. The first is inflammation; the observation of macrophages, lymphocytes, and multinucleated giant cells in areas such as interstitium and bronchial mucosa/submucosa is unlikely to be significantly affected by fixation artifact. While inflammatory cells in airways such as alveolar histiocytes, neutrophils, and lymphocytes can reasonably be expected to be displaced to a degree by formalin infusion, they are reported for their value in characterizing inflammatory response in the general region. We can also be confident about the diagnoses of fibrosis due to the confirmation of high collagen levels in the trichrome stains and structural changes in the H and E stains. Bronchitis diagnoses are also well supported by histopathological evidence. Vascular proliferation/neovascularization are similarly well supported by architectural features of histologic sections.

### Correlation of imaging and pathology findings

The primary imaging biomarker findings of this work are described in the text for group A and shown in Figs. 2 and 3 of the supplementary material for perfusion. Figure 5 shows the imaging biomarker findings for ventilation in group B subjects. Pathology results are visually demonstrated in Figs. 6, 7 and 8 (group A) and quantitatively listed in Figs. 9 and 10 (group B). Correlations between biomarker and pathology observations are described for direct and indirect radiation response as well as additional validation of ventilation biomarkers.

#### Direct radiation response: perfusion change

The perfusion imaging biomarkers from high-dose regions in both group A and B WMS suggest that radiation delivery induced vascular change and caused blood to leak from the vasculature. However, the slope of the change in the HU relationship in and out of the vessels was not 1 (Fig. 3 in the supplemental material). This non-unity slope, as discussed in our previous work^19^, suggested other mechanisms contributing to the rise in HU outside the vessels.

The pathology observations from groups A and B pigs support the main imaging hypothesis of vascular radiation-induced damage leading to red blood cell leakage from the vasculature. These are highlighted in Fig. 6 and in the max dose and contralateral columns of Fig. 10. In all but one pig, hemorrhaging was observed with proliferation of red blood cells into the lung parenchyma was observed (but not in the control slide). Red blood cell leakage combined with observations of vascular damage correlate with the radiation-induced decline in perfusion imaging biomarkers.

Figures 7, 8, 9 and 10 provide insight into some possible effects that could be related to rising HU values outside the vasculature. Collagen/Fibrosis was a consistent observation (seen in 8 of 10 pigs). Inflammation was noted for the two group A subjects, where fibrosis was not observed, and for all group B subjects. Additionally, several other subject-dependent effects presented as an HU increase. Inter-subject variation suggests some degree of individuality in overall response and the speed of disease progression. As shown in Fig. 9, the degree to which collagen/fibrosis was observed was inconsistent. For subjects that did not show significant collagen/fibrosis presence but did show inflammation, it’s possible that these subjects were experiencing a degree of pneumonitis, typically observed before the development of fibrosis. Further studies with a larger sample size are necessary to better understand the effects noted in Fig. 10 that only occurred in a fraction of subjects. Additionally, further studies with a more specific methods to verify functional changes would be useful in better understanding the exact mechanisms of change.

#### Direct radiation response: ventilation change

Physiological changes, such as alveolar wall thickening and edema, are known to be associated with reduced ventilation^57^. Fibrosis is also a commonly occurring post-RT toxicity that is associated with increased collagen and affects the lungs’ ventilation ability^58^. This study has identified correlations between post-RT changes in ventilation, as defined by CT-based biomarkers, and noted aberrations from normal lung tissue, as defined by histopathology. In high-dose regions, ventilation biomarkers indicated a ventilation decline of at least 6% (equivalently LER-N_*post*_*/*LER-N_*pre*_ < 0*.*94) in 9 of 10 subjects, with the tenth subject having a LER-N ratio of 0.95. 8 of 10 subjects had low ventilation values post-RT. Control points showed ventilation decline in no subjects, and only one subject had a low post-RT ventilation value. These biomarker results correlate very closely with 8 of 10 subjects presenting with increased collagen/fibrosis in highly irradiated regions but none in the contralateral lung. The agreement between these findings supports the hypothesized mechanism through which radiation directly impacts the lung’s local ventilation ability. Collagen presence reduces tissue elasticity (increasing stiffness) which corresponds to reduced tissue elasticity and therefore, expansion. The LER-N biomarker used in this work may be used to indirectly infer increased collagen and its location within the lung. The presence of inflammation in those without noted collagen, as described in the previous direct perfusion change section, are also relevant to the ventilation-based changes seen.

Specifically focusing on group B, the max dose region showed significantly (*p* < 0*.*05) more ventilation decline than the contralateral contour (highlighted by red and gray box plots in Fig. 5). The histologic diagnoses support this finding, with all subjects demonstrating chondrocyte necrosis, inflammation, and fibrosis, and a majority presenting with bronchitis and alveolar septa thickening. Contralateral control points showed minimal deviations from normal architecture, while the max dose region samples exhibit a variety of changes consistent with tissue damage and/or repair. Overall, CT-ventilation biomarkers and histopathology both show that radiation-induced damage occurs at the maximum dose point but not in the contralateral lung. It is hypothesized that along with collagen presence, further development of fibrosis, inflammation, bronchitis, and thickening of alveolar septa contribute to reduced ventilation, which was captured by imaging biomarkers.

#### Indirect radiation response: perfusion change

The imaging results from the indirect perfusion analysis indicated that vessels receiving doses below expected damage thresholds and supplied by highly irradiated vasculature (fed contours) demonstrated a significant reduction in blood flow post-RT. However, vessels receiving the same doses but not supplied by the highly irradiated vasculature did not, nor did the contralateral lung vessel analyzed.

The results reported in Figs. 9 and 10 support the perfusion-based findings observed in imaging. The majority of the subjects were diagnosed with fibrosis in the low dose-fed contour, but in the corresponding low-dose, not fed contour, no subjects experienced fibrosis. Inflammation appeared to be a more global effect appearing in 2–3 subjects in both fed and not fed contours. The fibrosis observed surrounding these vessels will cause constriction of the vasculature and thus reduce the flow of blood through those vessels in addition to thickening the tissue surrounding where gas exchange should occur. Additionally, previous studies have reported that vascular density is strikingly decreased in fibrotic areas of idiopathic pulmonary fibrosis which indicates a reduction in capillaries able to perfuse blood^58–60^.

The max dose contour, in addition to the inflammation and fibrosis already mentioned, experienced several additional effects that correlate with a reduction in perfusion. These effects varied by subject, but many appeared in the majority. Other observed effects included multinucleated giant cells, histiocytosis, type 2 pneumocyte hyperplasia, vascular proliferation, alveolar septa thickening, and proteinaceous fluid. As stated previously, the multinucleated giant cells are difficult to interpret alone, but due to the additional presence of histocytes, we can be confident these represent true inflammation in the region. The proteinaceous fluid as mentioned in the previous section is either edema or extravasation from vessels. Either would correlate with reduction in blood flow, but post-RT extravasation is more likely based on the observation of vascular proliferation in addition to the group A parenchymal observations. The indirect analysis points did not include enough surrounding parenchyma to thoroughly analyze if there was the infiltration of red blood cells, but the vascular proliferation observation indicates that there was damage to the vascular wall, which would allow for blood to leak outside of that vessel. This would further correlate with the observation of the low-dose-fed and no dose fed regions seeing reductions in contrast flow through the vessels^18^ because this blood would no longer be circulating.

One interesting note is that LDNF contours had multinucleated macrophages in all subjects, but in the LDF sections, this change was found in only two subjects. As stated previously, this diagnosis is difficult to interpret due to the formalin preservation methods used. It is difficult to determine if these observations are local to that region or if they were displaced from the max dose region during formalin infusion.

Finally, in addition to correlating with the main conclusions drawn from the previous imaging analysis, the histology diagnoses also correlate with some of the outliers that were previously discussed. In the imaging study, there was one pig that was reported to have no visible contrast flow in the low dose and no-dose-fed contours post-RT^18^. When analyzing this pig’s pathology, this pig was the only pig to present with vascular proliferation/neovascularization, and vascular necrosis in addition to the commonly observed fibrosis and inflammation diagnoses in the max dose contour. Vascular necrosis suggests ongoing damage to the vessels even at the 3 month timepoint. Vascular proliferation in regions of fibrosis is suggestive of a repair process replacing previously destroyed blood vessels. Both findings are evidence of reduced numbers of intact vessels perfusing this area. Neovascularization refers to the development of small microvasculature which has been shown in previous studies to result in a decrease in diffusing capacity consistent with a substantially reduced capillary volume^57^. This neovasculature also does not participate in pulmonary circulation thus reducing the flow of blood through the further downstream pulmonary vasculature^58,61^. The cause of this subject responding more severely is still unclear, but these added diagnoses further help to validate that the imaging observations are giving an accurate analysis of the changes in the function of the lung.

#### Indirect radiation response: ventilation change

Figure 5 shows ventilation reduction for the four indirect radiation response contours in regions of no dose (blue) and low dose (brown). Ventilation biomarkers were consistent in that not fed regions did not show ventilation decline for any subject. On the contrary, results in fed contours were inconsistent, showing a larger range of radiation response. One subject showed LDF ventilation decline while three showed NDF decline. Pathology results within indirect contours were also not consistent across subjects. Wallat *et al.*^17^ previously found a similar subject dependence, noting that fed regions showed greater ventilation decline than not fed regions, although not statistically significant. With this range in response, evaluating results on an individual subject basis provides insight.

In low-dose regions, ventilation biomarkers and pathology agree at all LDNF points since no subjects showed ventilation decline or collagen presence in these contours. The LDNF point also only showed a few histological diagnoses with minimal strength. For LDF contours, biomarkers and pathology agree in four out of five subjects. In subject A, no ventilation decline was noted, and mild chondrocyte necrosis was the only pathology finding. For subjects C-E, ventilation biomarkers indicated decline and had collagen presence, fibrosis, and inflammation noted among other histological diagnoses. The only disagreement in the LDF contour was in subject B. Ventilation biomarkers showed no decline in this subject. However, histology noted moderate fibrosis (confirmed by moderate-marked collagen presence) and minimal-mild chondrocyte necrosis in this contour.

For no dose regions, biomarkers and pathology agreed in a majority of contours. For not fed regions, ventilation biomarkers did not show decline in any subject. For pathology, subjects A and D showed both increased collagen and multiple minimal-mild strength diagnoses, including fibrosis and inflammation. Subjects B, D, and E did not have collagen presence noted and only a few minimal histological diagnoses, agreeing closer with ventilation biomarkers. Overall, NDNF findings agreed that subjects B, C, and E did not show significant evidence of ventilation decline. In subjects A and D, biomarkers showed no ventilation decline while pathology showed multiple diagnoses, albeit minimal-mild in strength. However, this may not necessarily be a disagreement between biomarkers and pathology; the specific histologic changes and their severities may not have significantly altered ventilation, i.e., minimal interstitial inflammation. For NDF contours, biomarkers and pathology agreed in four out of five subjects. Subjects A and B show no ventilation decline in this contour and have no collagen presence or ventilation-related diagnoses. Subject C showed the largest amount of ventilation decline and showed the most damage via pathology. This was the only subject with collagen presence and had seven diagnoses noted. Subject E showed ventilation decline and had diagnoses of inflammation and fibrosis. Subject D is the only subject where ventilation biomarkers disagreed with pathology in this contour. Ventilation biomarkers indicate decline but histiocytosis was the only diagnosis seen via pathology. Of note, analysis was only performed at a couple of locations at each point; something different could be seen 1 mm away.

Overall, pathology results correlate with ventilation biomarker observations in most subjects in all indirect radiation response contours. With four indirect points of interest across five subjects (a total of 20 contours), ventilation biomarker and pathology findings agreed in 16 cases. The four potential disagreements were in subject A (NDNF contour), subject B (LDF contour) and subject D (NDF and NDNF contours). In subjects A and B, these disagreements showed more damage via pathology than from ventilation biomarkers. For subject D, this was also the case for the NDNF contour, but the NDF contour had the opposite result, showing more decline from biomarkers than pathology. Biomarkers and pathology agree that strength of indirect ventilation decline was subject dependent. Further studies with more subjects and analysis in multiple indirect contours would help to better understand the nuances of this phenomena.

#### Ventilation-based validation

The parenchyma-centered ventilation points (HLV, LLV, HHV in Fig. 4) provided the best opportunity to validate ventilation biomarkers since their selection was based solely on pre- and post-RT ventilation values.

Of the ventilation contours, only the high-low ventilation region showed a functional decline after treatment as shown in Fig. 5. The mean decline in this contour was 12% and this point also had a low post-RT ventilation value by definition. These two biomarker-based findings of ventilation decline and low post-RT ventilation at the HLV point correlate with the pathology findings in Figs. 9 and 10. First, every subject had increased collagen at the HLV point; most had moderate or higher collagen, with levels very similar to those at the max dose point. Additionally, this point also had by far the greatest number of histological diagnoses for the ventilation points (Fig. 10). All seven diagnoses seen at ventilation points were seen in at least two subjects for this point. Subject A had the largest amount of biomarker-defined ventilation decline (16%), the highest levels of collagen, and the most damage as noted by histological diagnoses. Subjects B-E had similar ventilation decline to one another and similar levels of collagen and histologic diagnoses. Overall, CT-ventilation biomarkers and histopathology closely correlate in the high-low ventilation region of interest. Biomarkers demonstrated ventilation decline and low post-treatment ventilation while histopathology showed consistent tissue damage in this region through multiple mechanisms. This can also be seen in the radiation response points of interest.

According to ventilation biomarkers, the high–high ventilation region showed no decline in ventilation following treatment (Fig. 5) and maintained high ventilation values. Histopathology supports both of these results. First, only two subjects had very minimally increased blue staining on Masson’s Trichrome stained sections (indicating collagen) at small alveoli at the HHV point as in Fig. 9. Both findings were listed as suspect artefacts of fixation by the performing pathologist. Specifically, slowed/incomplete fixation can affect how staining looks. High-high ventilation points also showed the fewest histologic diagnoses in Fig. 10, on par with that of the contralateral lung. Subjects A-B showed no diagnoses. Subject C only showed suspicious multinucleated macrophages. Subject D showed minimal fibrosis and mild inflammation, agreeing with the minimal collagen presence noted from the trichrome stain. Subject E showed just minimal histiocytosis. No ventilation decline, high post-RT ventilation values, and no consistent collagen presence or diagnosis indicates that ventilation biomarkers and histopathology agree at the high-high ventilation point. This region of healthy tissue was marked as highly ventilated from imaging biomarkers and did not show tissue damage when evaluated with histopathology.

The final ventilation-based point, low-low ventilation or LLV, is more nuanced. This point had low ventilation in healthy subjects before any radiation was delivered. As demonstrated in Fig. 5, the ventilation value was not reduced by treatment, but the point still had a low ventilation value post-RT. For histopathology results, collagen is seen somewhat consistently at this point; 4 of 5 pigs have some amount of collagen, most being minimal. Comparatively, this point shows less collagen than HLV but more than HHV. Similarly, the number of histologic diagnoses seen in LLV in Fig. 10 falls between those for HHV and HLV. Only chondrocyte necrosis was seen in all subjects, but a subset did have inflammation and fibrosis. At the LLV point, histopathology results generally support those of ventilation biomarkers. In terms of ventilation decline, the LLV point shows less histologic damage than points that did show decline (HLV, MD and LDF/NDF in some subjects). However, the point does show more collagen and diagnoses than HHV and CON points.

Interpretation of the low ventilation point suggests how initially low-ventilating tissue may differ from highly ventilated tissue or tissue that is only poorly ventilated after being damaged from radiation. Of note, this contour showed no bronchitis, compared to bronchitis in most subjects at the MD and HLV points, which consistently showed pre- to post-RT ventilation decline. This result suggests that bronchitis (up to bronchial ulceration in some cases) is potentially a radiation-induced change, since not seen in low-low ventilation regions that didn’t decline with treatment. The strength of fibrosis and inflammation in this contour is also reduced to those in the MD and HLV contours. Chondrocyte necrosis appearing in all subjects also may be indicative of low-ventilating tissue, radiation-induced or not. As originally hypothesized, the low-low ventilation region shows damage amounts between high-high ventilation and high-low ventilation regions. Exact mechanisms cannot be concluded from this study but deserve to be explored in future work.

Ventilation wise, by evaluating regions of consistently high ventilation, consistently low ventilation, and high reduced to low ventilation, CT-ventilation biomarkers correlate closely with histopathology analysis. Regions of high ventilation showed minimal damage or change from normal histologic findings. Regions where ventilation was reduced by treatment showed consistent evidence of lung damage. Low ventilation regions were in between, with some damage. Combining these results with those for described previously radiation response points can be used to comment on overall biomarker and pathology agreement. For nine contours in the five group B pigs, totaling 45 evaluation points, biomarkers and pathology agreed at 41 points (91%) and disagreed at only 4 points (9%). An over 90% agreement rate between biomarker metrics and histopathology findings provides strong evidence that Jacobian-based ventilation biomarkers are representative of underlying pathology changes related to ventilation function. To our knowledge, no previous work has correlated CT-ventilation with pathology measures to demonstrate direct and indirect radiation response. This novel finding highlights the accuracy with with CT-ventilation can represent tissue changes affecting ventilation.

### Study limitations

In this study, CT-based ventilation and perfusion biomarkers were compared to histopathology measures in two groups of swine. After performing experiments in the first group of swine, experimental design was adapted and improved for the second group of swine. The improved methodology between groups addressed some important study limitations. Compared to group A, group B subjects had a more superiorly placed dose distribution, allowing analysis of indirect radiation response regions downstream of the dose distribution. This dose distribution placement, combined with the improved method of localizing ROIs, allowed more points to be analyzed in group B WMS and expanded study scope. Finally, more detailed histopathology was performed at each point, better elucidating local effects and deviations from normal tissue properties.

Even with experimental design improvements between WMS groups, several general study limitations remain. The subject sample size limits applicability of results and further studies in larger cohorts are necessary to increase confidence. Studies in larger subject cohorts would be particularly valuable for better understanding indirect radiation response, which was found to be a subject-dependent effect. Additionally, perfusion and ventilation metrics were derived from images acquired on one CT scanner with narrowly defined methodologies. Results cannot be generalized to biomarkers acquired using different image acquisition or post-processing/derivation techniques without additional experiments. In terms of the pathology, the largest limitations are the restricted tissue sections and evaluation by one pathologist. As previously mentioned, 1 inch tissue sections were used to evaluate pathology, but different findings could have been observed nearby. Tissue sectioning and analysis was performed by a single pathologist who was not masked to the pathology specimen. Objectivity and thoroughness of evaluation could be improved by involving multiple pathologists who are masked to the ROIs.

### Clinical implications

With the pathology validating the observations from both perfusion and ventilation imaging, several opportunities to expand the use of imaging biomarkers are introduced. In general, non-invasive biomarkers that can assess disease progression or prognoses are valuable and can help to guide interventions. The current gold standard of lung function testing is using pulmonary function tests. However, these methods struggle in that they have large variance and are effort-dependent global metrics^4^. Imaging biomarkers present an opportunity to extract regional information that is more robust to the patient’s condition at pre-RT. This regional aspect has particular use in functional avoidance radiation therapy, as described in the introduction, in which treatment planning is optimized to avoid dosing regions of high function with the goal of sparing functional lung and improving patient quality of life post-RT. To execute functional avoidance, detailed dose response models need to be developed from imaging to decide which regions to avoid. The work presented here validates two metrics that encompass the main components of lung function (ventilation and perfusion). With these methods validated, we can use them to proceed with studying the nuanced radiation-induced changes in lung function and develop more advanced models.

## Conclusions

Imaging biomarkers play a vital role in the diagnosis and tracking progression of disease in the lung. When validated, they offer regional functional information that cannot be captured by the existing gold standard and are robust to subject effort. The results of this work present previously developed perfusion and ventilation imaging biomarker techniques and present the radiation-induced changes in those metrics combined with correlating pathology to validate the observations presented previously using a novel porcine model. With these results, the imaging techniques were shown to measure radiation-induces physiological changes in the lung and may be used in further studies to understand more nuanced radiation-induced behaviors.

## Supplementary Information

Supplementary Information.

## Data Availability

The original contributions presented in the study are included in the article/supplementary material, further inquiries can be directed to the corresponding author.
