# Supplementary material for: Validation of CT-based ventilation and perfusion biomarkers with histopathology confirms radiation-induced pulmonary changes in a porcine model
Source: Sci Rep. 2023 Jun 9;13:9377. doi: 10.1038/s41598-023-36292-0 (PMC10256800; doi:10.1038/s41598-023-36292-0)
Supplement: Supplementary file 1 — Supplementary Information. [file 41598_2023_36292_MOESM1_ESM.pdf]

1 Details regarding the use of Wisconsin Miniature Swine

1.1 Novelty of the WMS^{TM} Model Compared to conventional Swine Models

The WMS^{TM} at the size used in our study (matching human adult lungs) were pigs in their early adulthood. If we had used conventional breed of swine as previous studies have done, the pigs would have been about 3 months of age (in order to approximate human lungs). Given that pigs reach sexual maturity at 5 months of age, a 3-month old pig is equivalent to a pre-pubescent human child at 6-8 years of age. A conventional swine at this age has a dramatic rate of development where tissue remodeling and size changes are very rapid. It's ability to heal is incredible like a human child. That is, the pigs' response to radiation damage (i.e., pathophysiology) would probably not mimic that of a human adult. The WMS^{TM} on the other hand allowed us to more closely model the pathophysiology observed in a human adult. A 14 month old WMS^{TM} is close to a human in the mid-late twenties.

1.2 Detailed Description of Methods

1.2.1 Indwelling Catheter Placement

Dantrolene was administered prophylactically (5.9 mg/kg) in a small amount of feed to prevent against malignant hyperthermia. Anesthesia was administered using an injectable Telazol/Xylazine cocktail (3 mg/kg of Telazol and 1.5 mg/kg of Xylazine). Gas anesthesia was then administered via a nose cone (isoflurane at 2%). The subjects' necks were shaved and scrubbed bilaterally. Surgical depth of anesthesia was confirmed by testing toe pinch response and palpebral reflex. An indwelling central venous catheter was placed percutaneously in the vena cava (placement confirmed by fluoroscopy) and secured in place with suture. The catheter was sutured to the skin twice using finger-trap suture technique as well as using 2 butterfly clamps. The subject was outfitted with a pocketed catheter jacked, placed over a spandex shirt.

Prior to catheter placement 20 mL of blood was collected for serum and plasma samples. A 30 mL 0.9% saline flush was administered and anesthesia was discontinued. The subject was moved to the recovery pen for 2.5 hours before being returned to housing and given a feed ration.

1.2.2 Each imaging and fraction delivery session

Dantrolene was administered prophylactically (5.9 mg/kg) in a small amount of feed to prevent against malignant hyperthermia Anesthesia was administered using an injectable Telazol/Xylazine cocktail (3 mg/kg of Telazol and 1.5 mg/kg of Xylazine). Gas anesthesia was then administered via a nose cone (isoflurane at 2%). The subject was intubated and placed in a transport cart to be transported to the CT suite.

Once in the CT suite, Propofol was administered again (2.4 mg/kg) and Telazol (1.2 mg/kg) was administered in the left hind leg. The subject was mechanically ventilated at 15 breaths per minute and 1000 mL/breath. Mechanical ventilation of subject was periodically adjusted to appropriately maintain SPO2 and ETCO2 while allowing for desired image acquisition and irradiation. For all 4DCTs, ventilation was reduced to 8 breaths per minute and for all contrast enhanced CTs the subject was placed in an inspiration breath hold with a 1000 mL tidal volume. Two contrast-enhanced scans were performed. Each contrast scan involved injection of 80 mL of Omnipaque 300 followed by 50 mL of saline administered intravenously.

Once imaging was complete, ventilation, gas anesthesia, and saline administration were discontinued while the subject was transported from the CT suite to the MRI-Guided LINAC vault. Once there, ventilation was resumed at 750 mL/breath and 8 breaths per minute with an inspiratory: expiratory ratio of 2:1. Irradiation treatment of the lung was performed to deliver 12 Gy to the left lobe targeted at a vessel bifurcation. Once irradiation was complete, anesthesia was discontinued and the subjects were weaned off mechanical ventilation. The subjects were transported to housing where they were extubated.

1.2.3 Description of the Dynamic Perfusion 4DCT

The dynamic 4DCT images were acquired over the central 15 cm of the lung as 80 ml of iodine contrast (Omnipaque 300) was injected at a rate of 5 ml/sec. The acquisition consisted of repeated scanning of the same volume at 1.5 sec intervals for 45 seconds and then every 4.5 seconds until the contrast had washed out of the lung. The Dynamic CT given to the pigs is not given to humans as part of clinical workflow. We believe this scanning protocol is a better indication of perfusion than the standard blood volume dual energy scan because it scans the same volume over a period of time as contrast flows in and out of the vasculature as opposed to capturing a snapshot at one time point of where the contrast is in the lung.

1.3 Animal Care During Study

All WMS^{TM} were housed on-site in facilities managed by the Biomedical Research Model Services group at the University of Wisconsin. Facilities undergo frequent inspection by the university Animal Care and Use Committee (ACUC) to ensure ethical treatment of animal subjects.

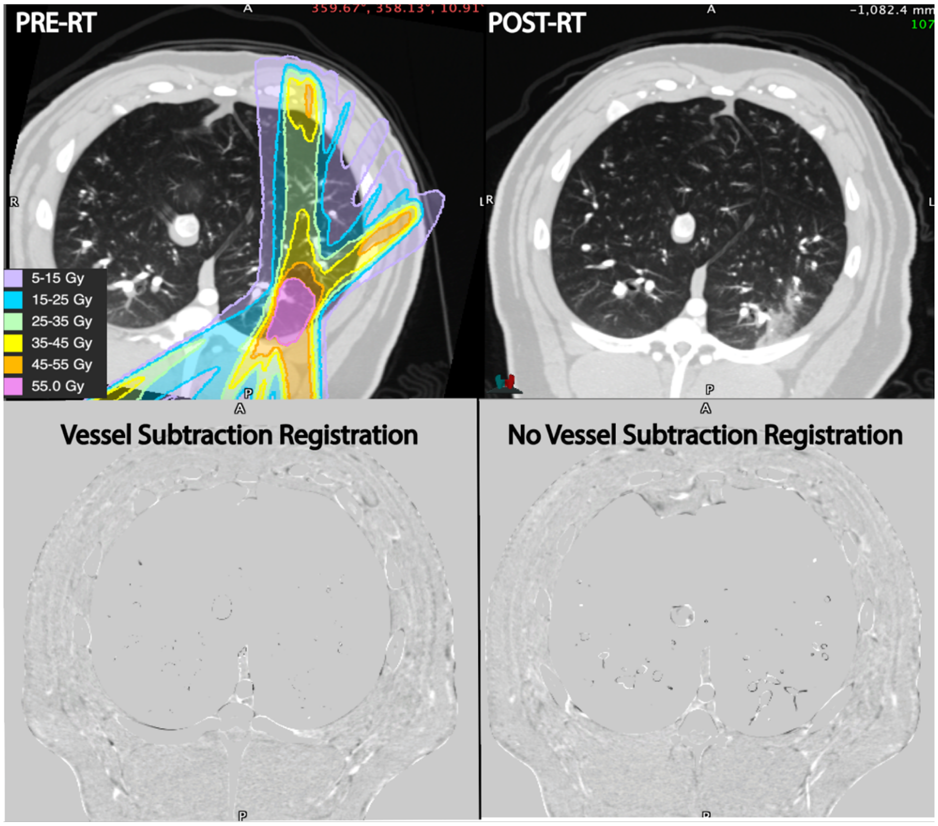

Figure 1. Justification for removal of vasculature showing the error in registration around regions of high vasculature.

2 Justification of vasculature removal procedures

Figure 1 illustrates the benefit to vasculature subtraction in the swine model. The bottom left image shows the post-RT scan deformably registered to the pre-RT scan and placed on top of the pre-RT scan in inverse grayscale. The bottom right shows the same however this image did not have vasculature subtracted prior to registration. In the image without vasculature registration it is clear there are errors in the registration where, particularly at the locations of vasculature, the registration algorithm does not perfectly align the post-RT image into the coordinate system of the pre-RT image. The differences in the inferior left lobe are in locations where dose was delivered and where we performed our analysis. Particularly, looking at the dose distribution on the upper left image, it's clear there is a sharp gradient In this region, therefor the error in registration would have skewed results since voxels containing vasculature could have been mislabeled into a different dose bin in the post-RT analysis than it was in in the pre-RT image. It is important to note we do not expect the registration error is in fact increased by the inclusion of the vasculature, we expect the opposite. However, the errors in registration are most noticeable around the vasculature which is why they are so noticeable in the bottom right.

3 Perfusion Imaging Biomarker Findings

Previously published results of direct and indirect radiation response of perfusion imaging biomarkers are highlighted in Figures 2-3.

4 Anatomical Measurements for Pathology: Group B WMS

In group B WMS, regions of interest were localized spatially by making airway measurements on post-RT imaging and applying the measurements to post-mortem lungs. Figure 4 shows an example of Slicer-based fiducial marking and resultant measurements. Figure 5 shows an application of image-based measurements to post-mortem lungs.

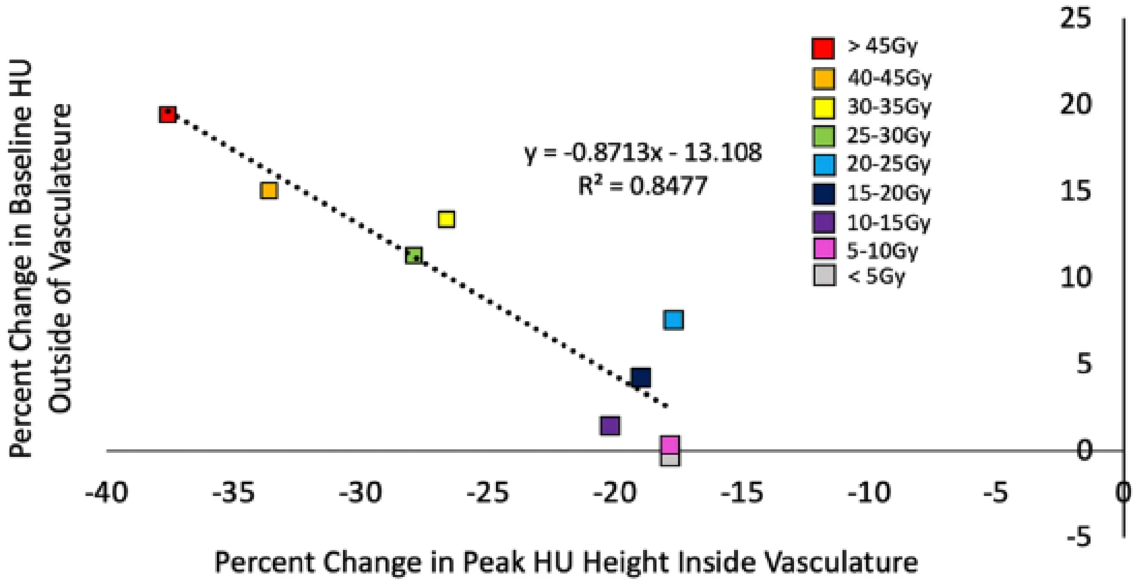

Figure 2. Reduction in HU inside the vessels corresponds to an increase outside the vessels. Each point on the line corresponds to a different dose bin analyzed and is the average of all WMS subjects. A strong correlation between the measurements exists for doses above 25 Gy. Adapted from Wuschner et al. [2] with permission.

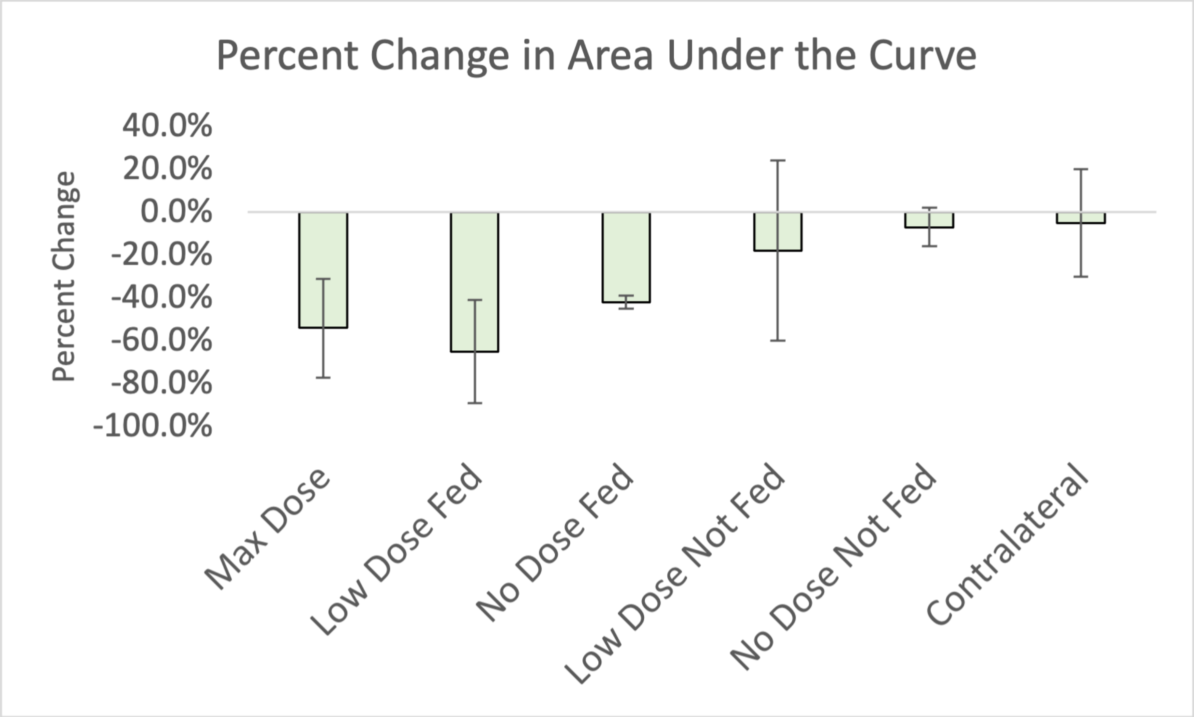

Figure 3. Percent change in area under the curve for each contour analyzed. Each bar represents the average of the five subjects (or four in the case of the no-dose fed contour). Error bars are the standard deviation of the percent changes in subjects. Statistically significant reductions were observed in all fed contours while not fed and contralateral contours did not. Copied from Wuschner et al. with permission [1].

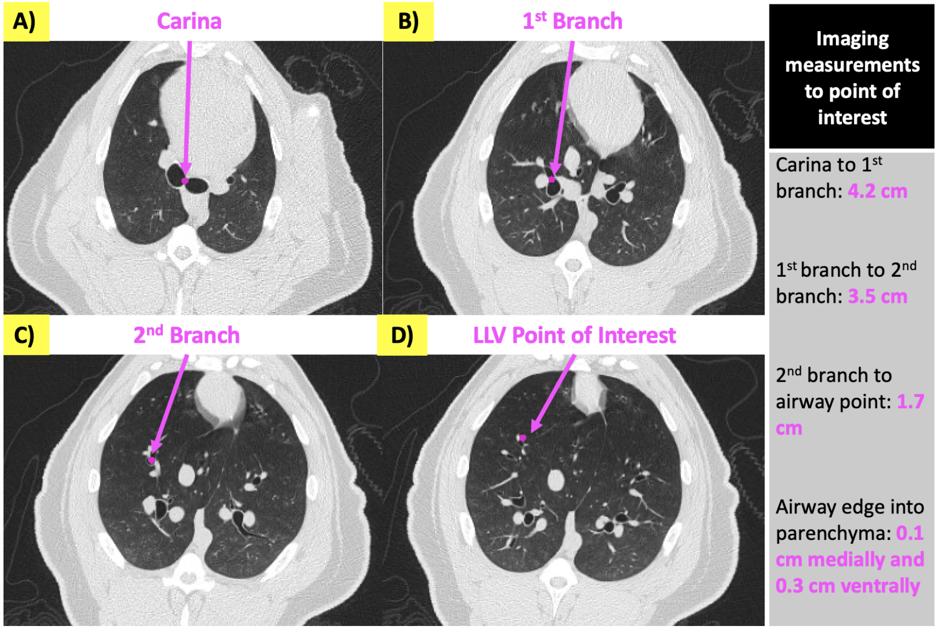

Figure 4. 3D Slicer fiducial markings (pink points) were used to measure distances along airways. A) Measurements started at the carina. B-C) Distances to airway branch points were measured. D) The point of interest is marked. Distances between fiducials are listed in the right-hand table. Figure 5 shows corresponding measurements in tissue.

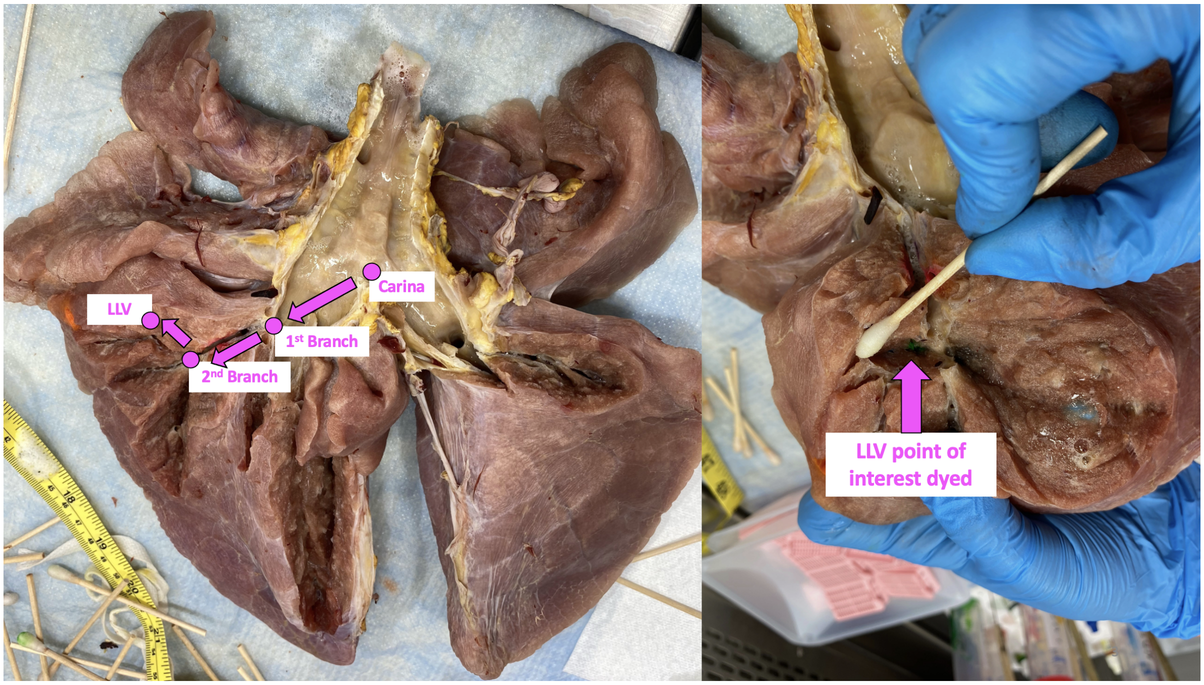

Figure 5. Measurements from Figure 4 are followed to reach a point of interest, which is dyed for marking purposes.

References

1. A. E. Wuschner, M. J. Flakus, E. M. Wallat, J. M. Reinhardt, D. Shanmuganayagam, G. E. Christensen, and J. E. Bayouth. Measuring Indirect Radiation-Induced Perfusion Change in Fed Vasculature Using Dynamic Contrast CT. *Journal of Personalized Medicine*, 12(8), 2022.

2. A. E. Wuschner, E. M. Wallat, M. J. Flakus, D. Shanmuganayagam, J. Meudt, G. E. Christensen, J. M. Reinhardt, J. R. Miller, M. J. Lawless, A. M. Baschnagel, and J. E. Bayouth. Radiation-induced Hounsfield unit change correlates with dynamic CT perfusion better than 4DCT-based ventilation measures in a novel-swine model. *Scientific Reports*, 11(1), 12 2021.
